# Supplementary material for: Weighted multiple testing procedures in genome-wide association studies
Source: PeerJ. 2023 Jun 15;11:e15369. doi: 10.7717/peerj.15369 (PMC10276986; doi:10.7717/peerj.15369)
Supplement: Supplemental Information 1 [file peerj-11-15369-s001.pdf]

SUPPLEMENTARY MATERIALS FOR ”WEIGHTED MULTIPLE TESTING PROCEDURES IN GENOME-WIDE ASSOCIATION STUDIES”

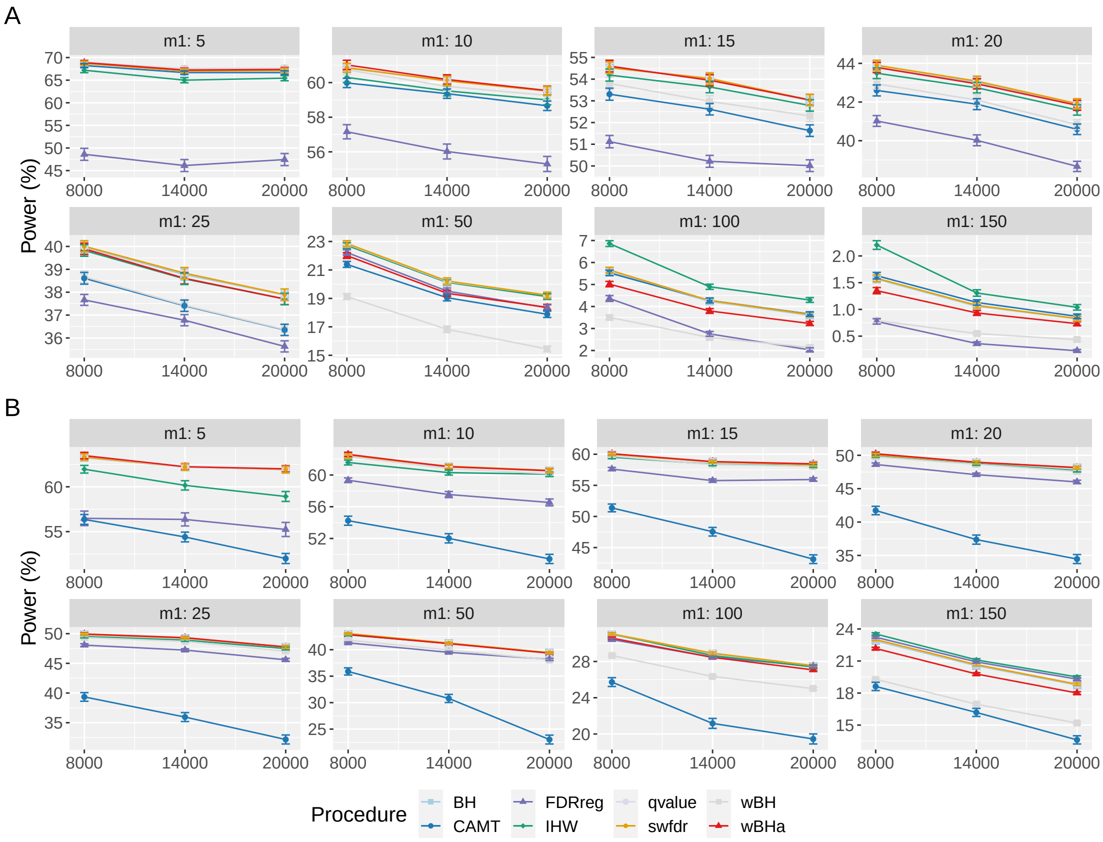

Figure S1. Overall power comparison in scenario 2, with independent markers ($\rho = 0$), for different m and m_1 values. Panels A and B display results for quantitative and binary traits, respectively. Vertical bars illustrate standard errors.

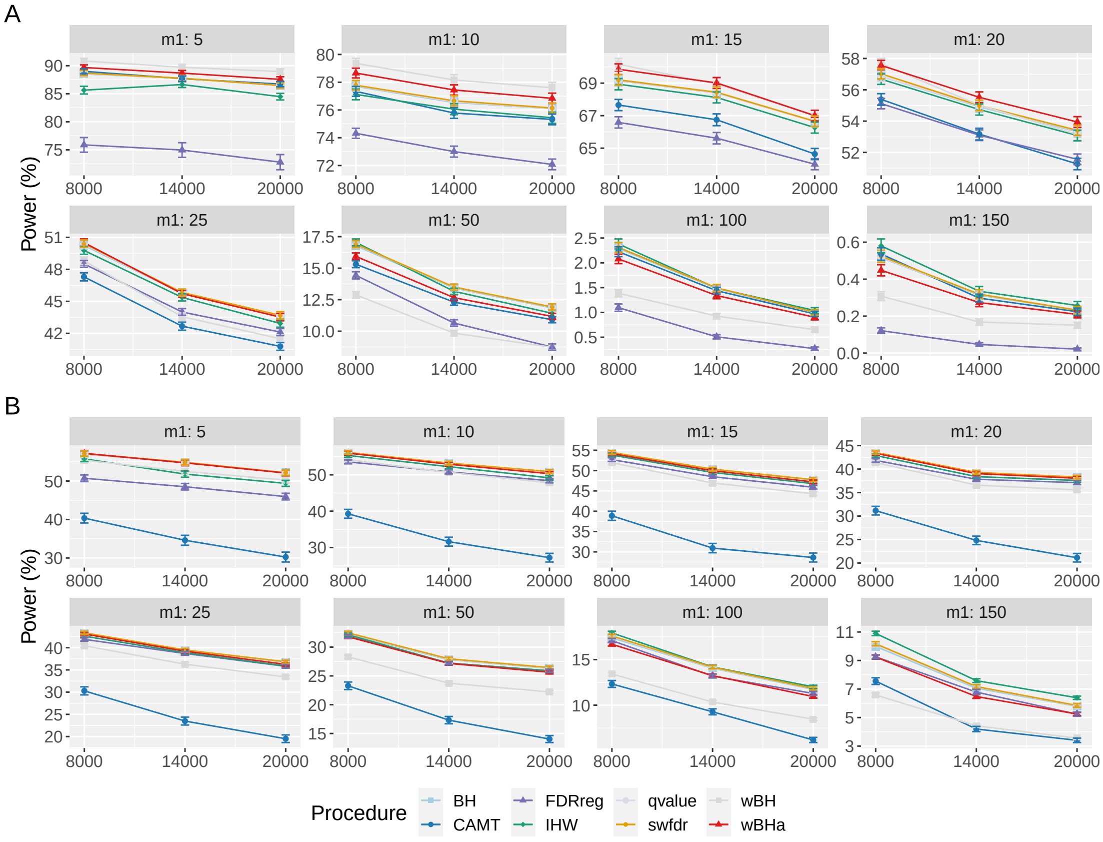

Figure S2. Overall power comparison in scenario 3, with independent markers ($\rho = 0$), for different m and m_1 values. Panels A and B display results for quantitative and binary traits, respectively. Vertical bars illustrate standard errors.

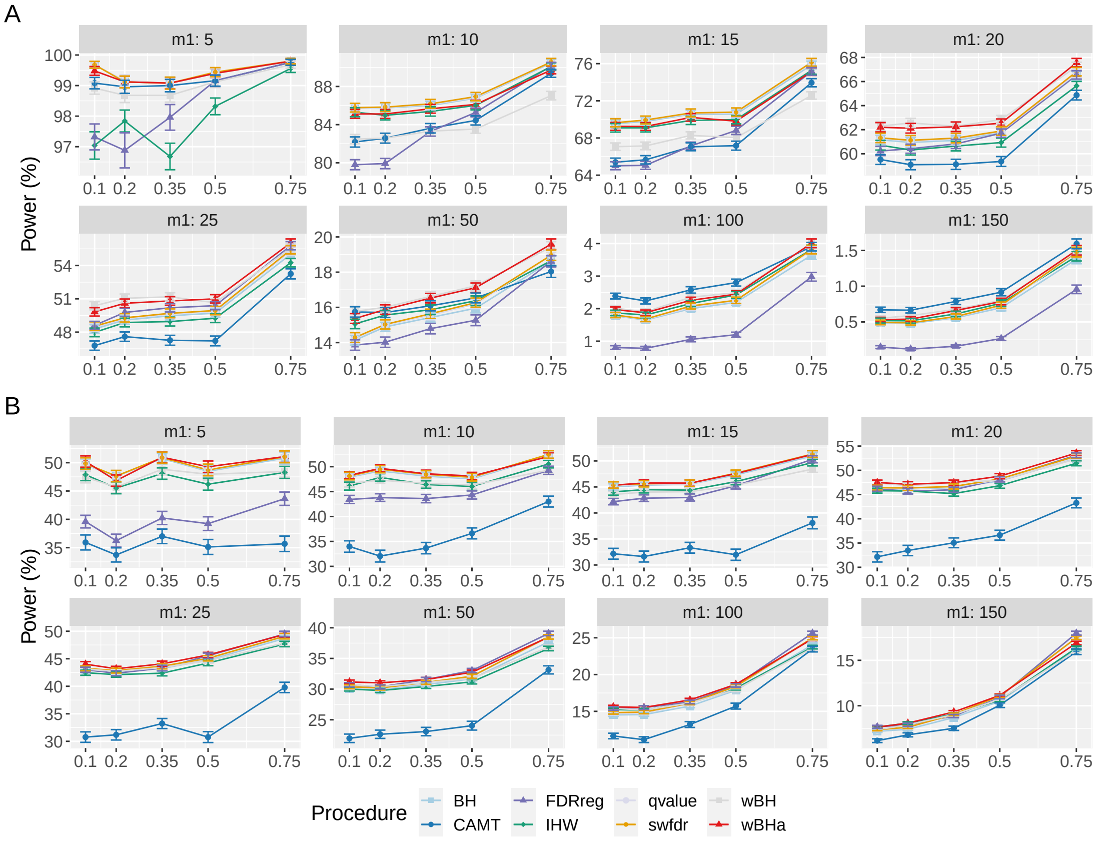

Figure S3. Overall power comparison in scenario 1, with correlated markers, for different ρ and m_1 values with $m = 8000$. Panels A and B display results for quantitative and binary traits, respectively. Vertical bars illustrate standard errors.

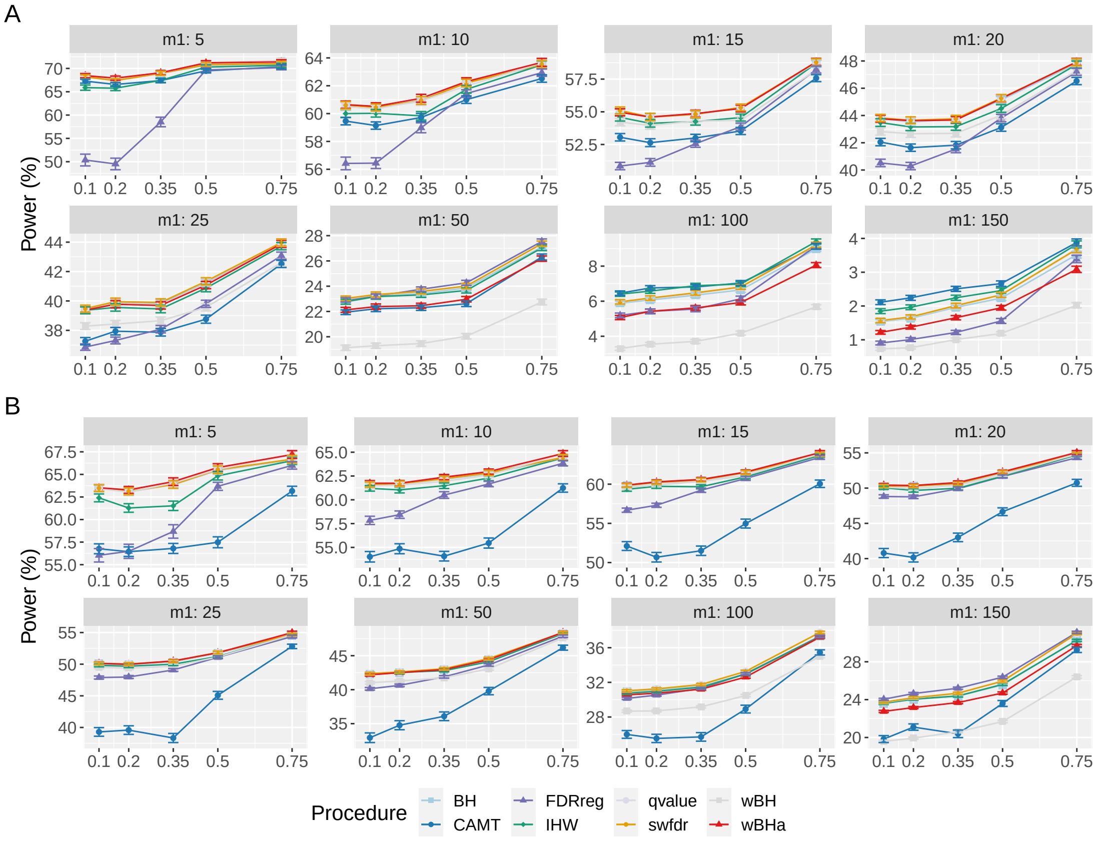

Figure S4. Overall power comparison in scenario 2, with correlated markers, for different ρ and m_1 values with $m = 8000$. Panels A and B display results for quantitative and binary traits, respectively. Vertical bars illustrate standard errors.

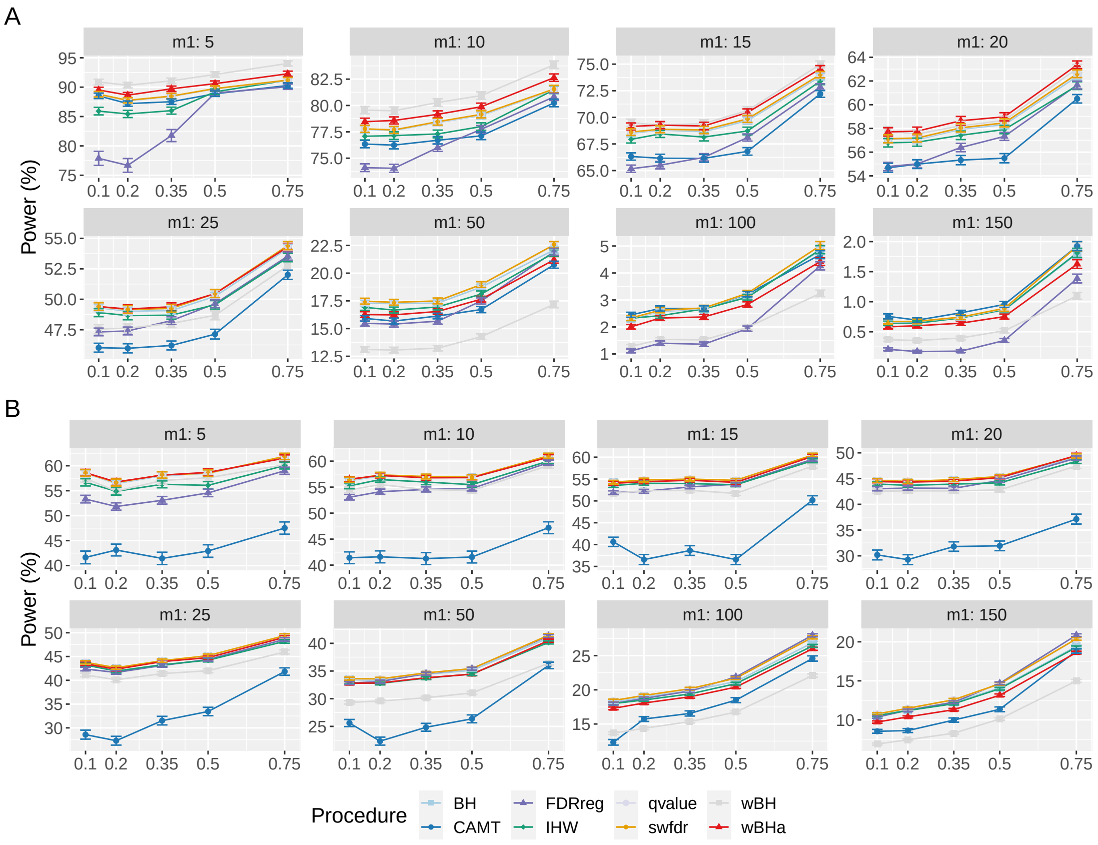

Figure S5. Overall power comparison in scenario 3, with correlated markers, for different ρ and m_1 values with $m = 8000$. Panels A and B display results for quantitative and binary traits, respectively. Vertical bars illustrate standard errors.

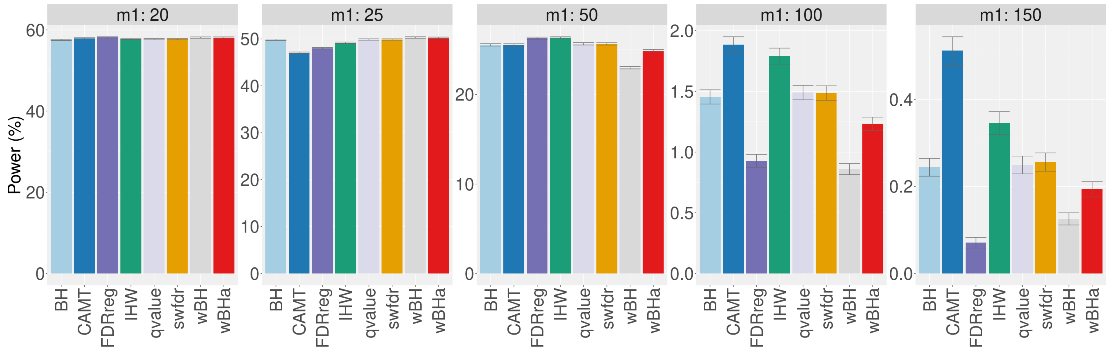

Figure S6. Overall power comparison in scenario 2, with simulations based on real data, for different m_1 values. Vertical bars illustrate standard errors.

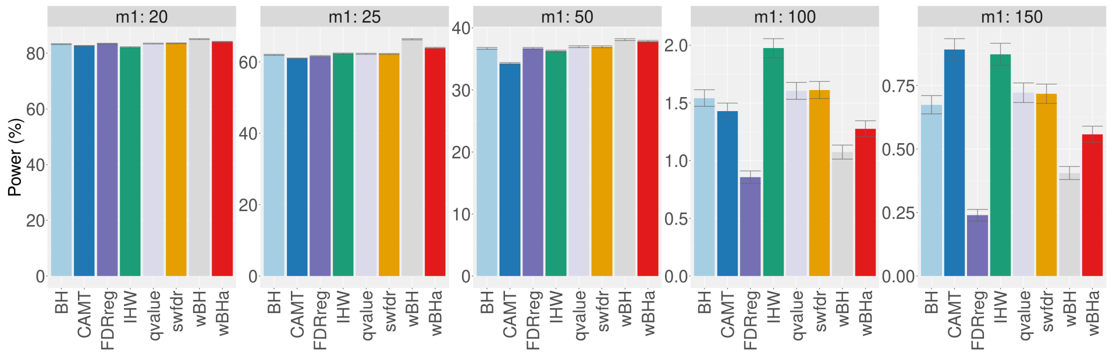

Figure S7. Overall power comparison in scenario 3, with simulations based on real data, for different m_1 values. Vertical bars illustrate standard errors.

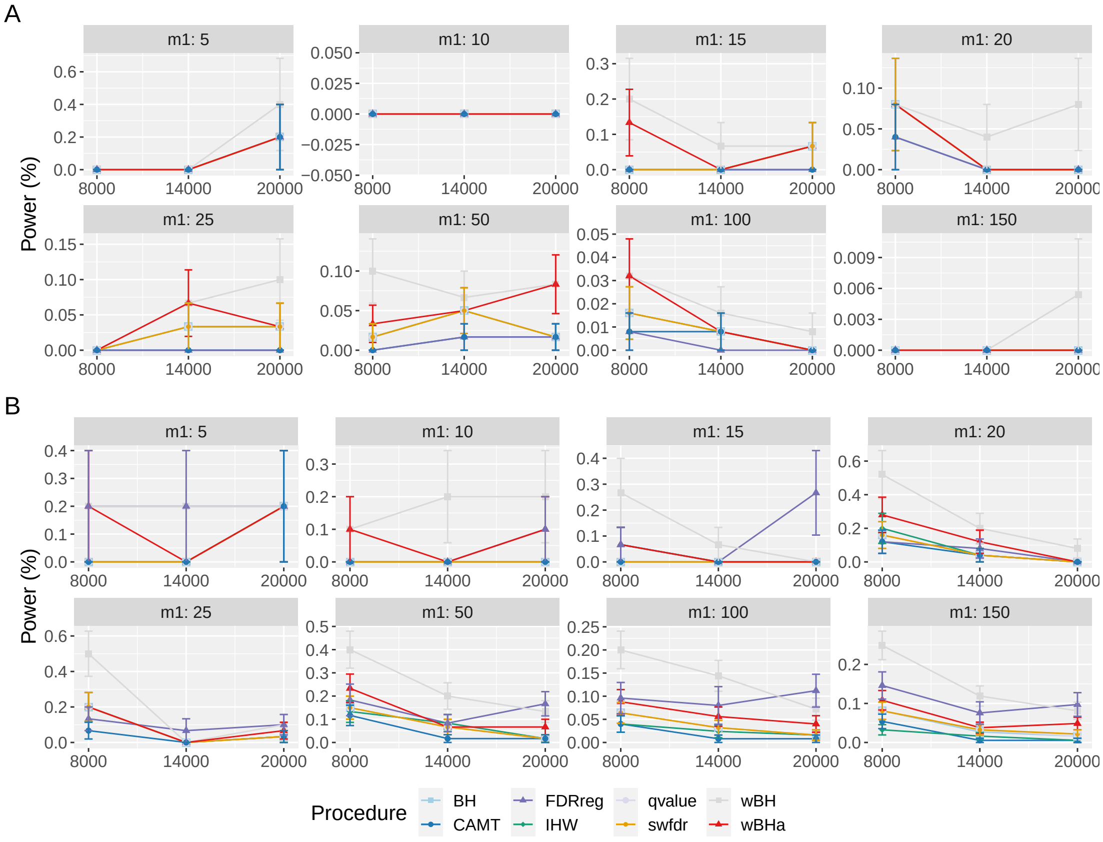

Figure S8. Power comparison in subgroup of rare variants in scenario 2, with independent markers ($\rho = 0$), for different m and m_1 values. Panels A and B display results for quantitative and binary traits, respectively. Vertical bars illustrate standard errors.

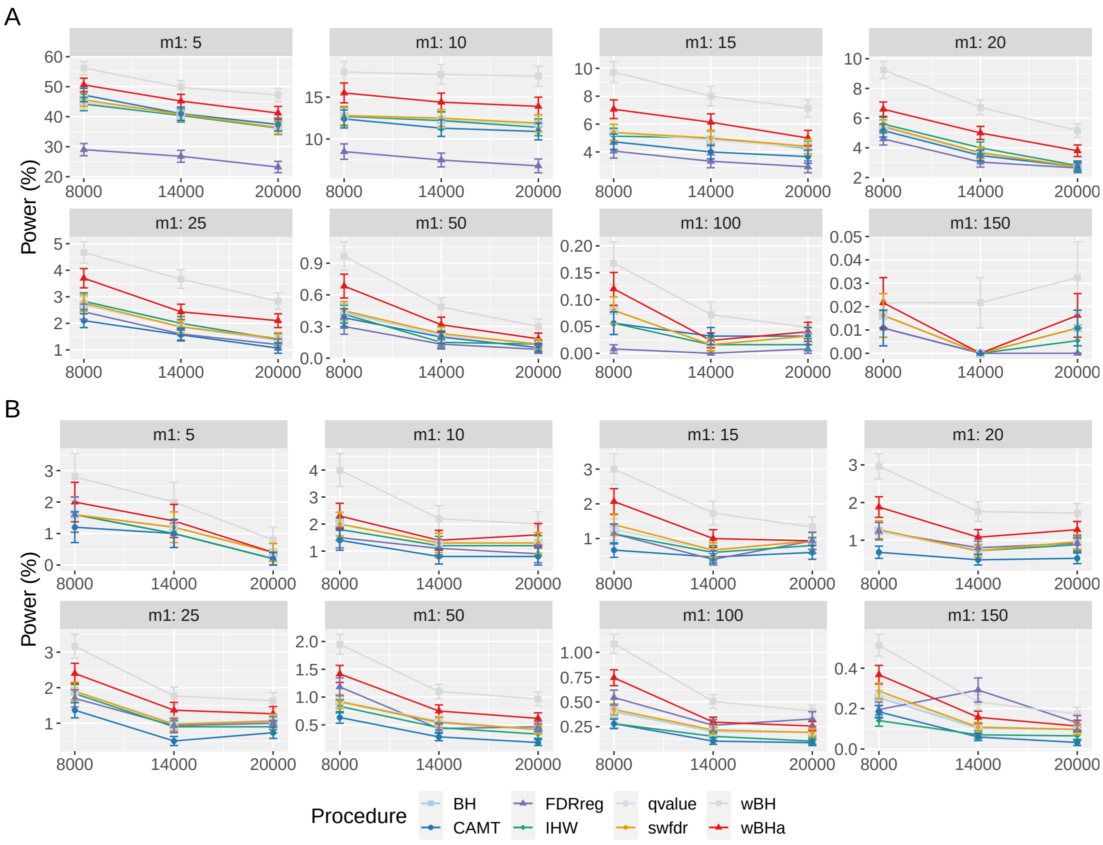

Figure S9. Power comparison in subgroup of **rare variants** in **scenario 3**, with **independent** markers ($\rho = 0$), for different m and m_1 values. Panels A and B display results for quantitative and binary traits, respectively. Vertical bars illustrate standard errors.

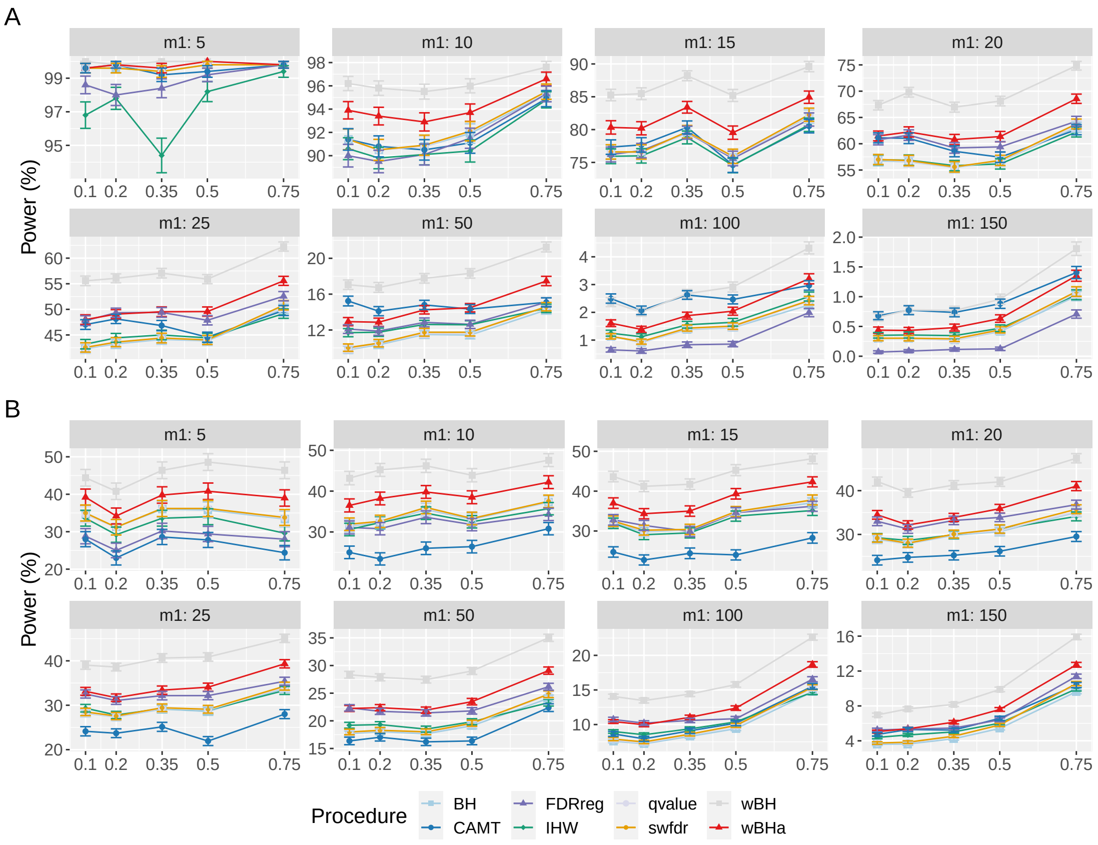

Figure S10. Power comparison in subgroup of **rare variants** in **scenario 1**, with **correlated** markers, for different ρ and m_1 values with $m = 8000$. Panels A and B display results for quantitative and binary traits, respectively. Vertical bars illustrate standard errors.

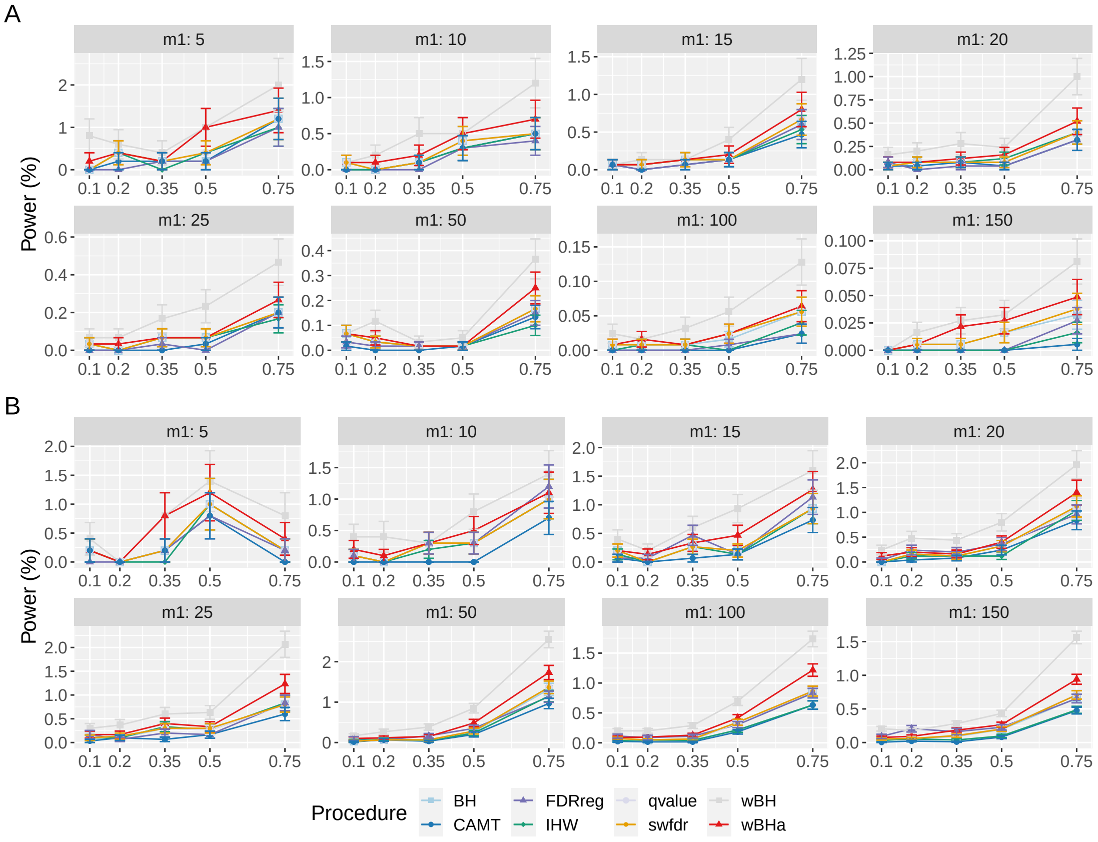

Figure S11. Power comparison in subgroup of **rare variants** in **scenario 2**, with **correlated** markers, for different ρ and m_1 values with $m = 8000$. Panels A and B display results for quantitative and binary traits, respectively. Vertical bars illustrate standard errors.

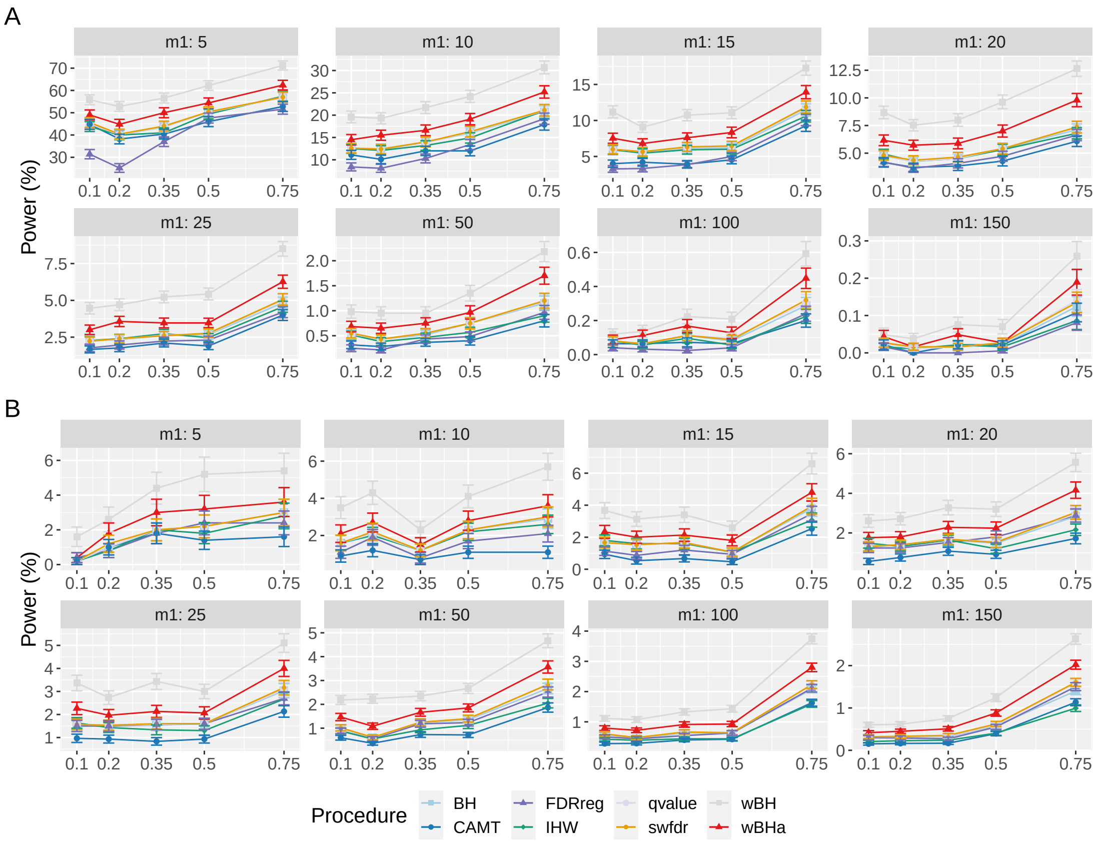

Figure S12. Power comparison in subgroup of **rare variants** in **scenario 3**, with **correlated** markers, for different ρ and m_1 values with $m = 8000$. Panels A and B display results for quantitative and binary traits, respectively. Vertical bars illustrate standard errors.

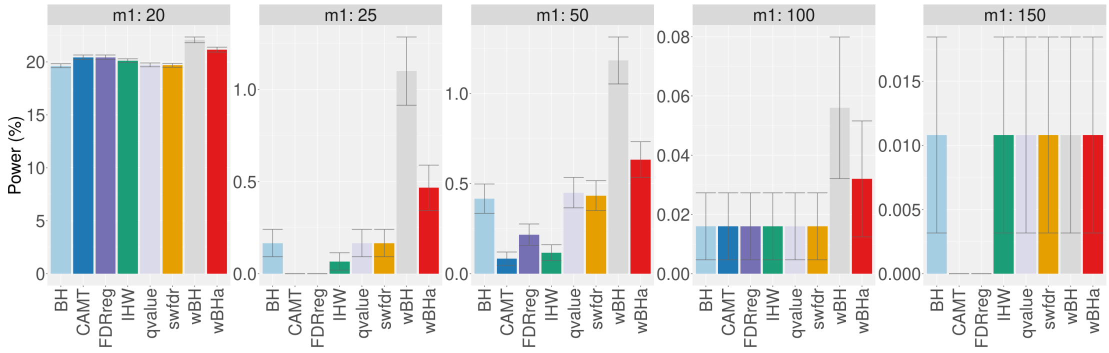

Figure S13. Power comparison in subgroup of **rare variants** in **scenario 2**, with simulations based on **real data**, for different m_1 values. Vertical bars illustrate standard errors.

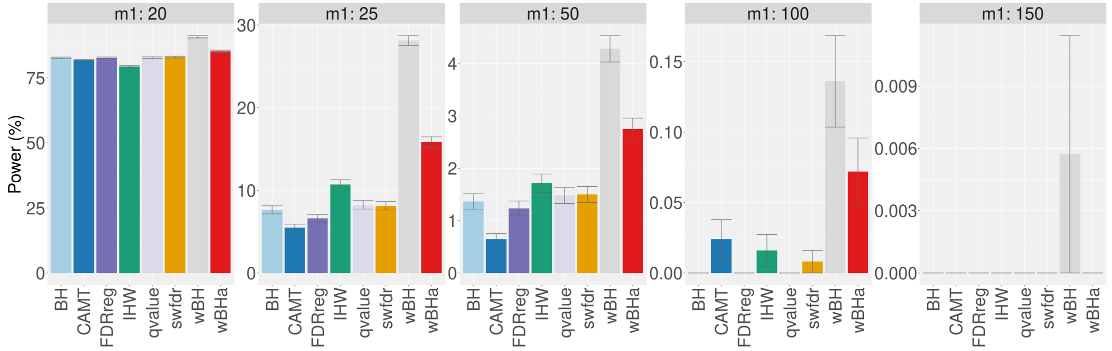

Figure S14. Power comparison in subgroup of **rare variants** in **scenario 3**, with simulations based on **real data**, for different m_1 values. Vertical bars illustrate standard errors.

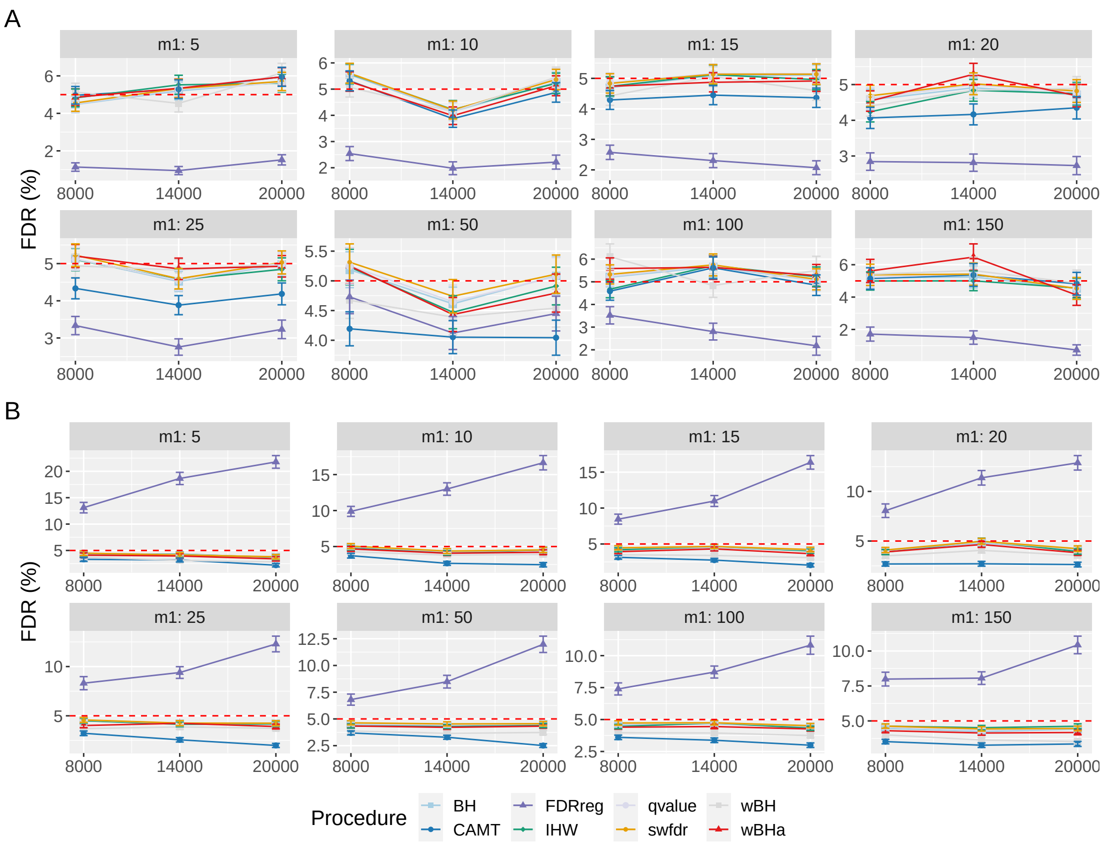

Figure S15. **FDR** comparison in **scenario 2**, with **independent** markers ($\rho = 0$), for different m and m_1 values. Panels A and B display results for quantitative and binary traits, respectively. Red dashed line corresponds to target FDR level (5%). Vertical bars illustrate standard errors.

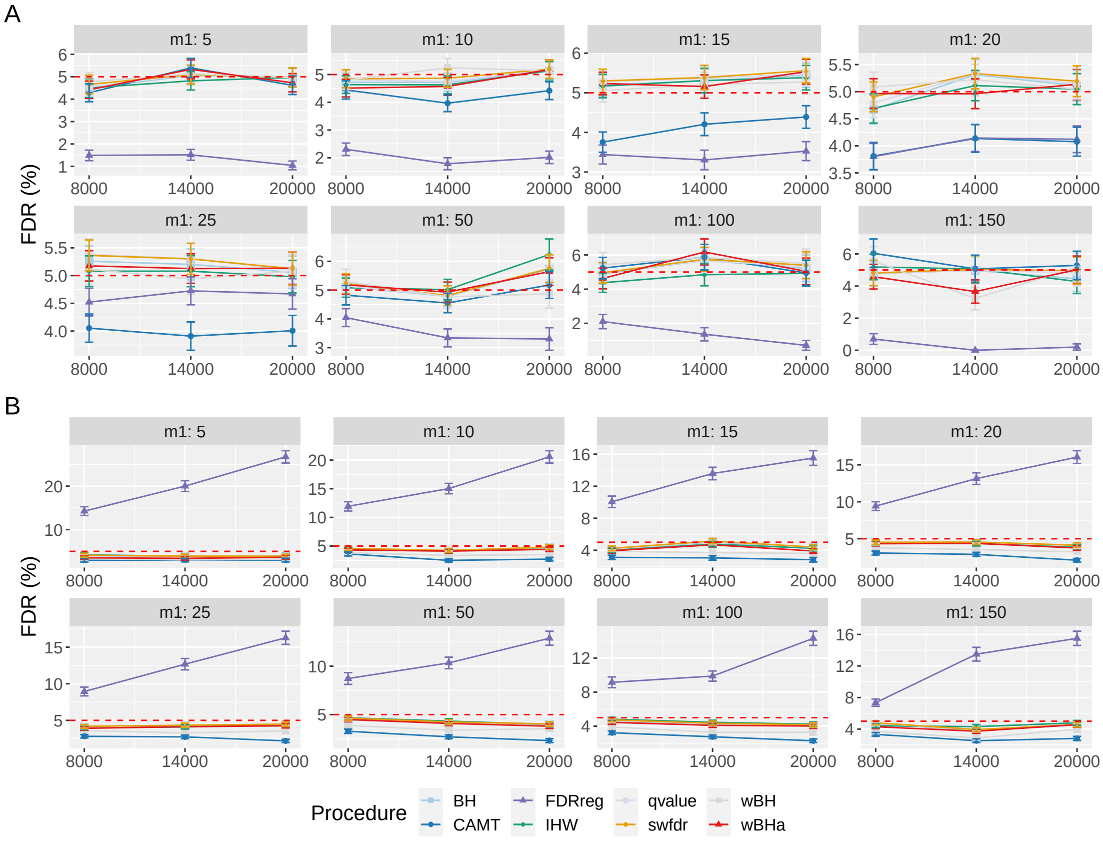

Figure S16. FDR comparison in scenario 3, with independent markers ($\rho = 0$), for different m and m_1 values. Panels A and B display results for quantitative and binary traits, respectively. Red dashed line corresponds to target FDR level (5%). Vertical bars illustrate standard errors.

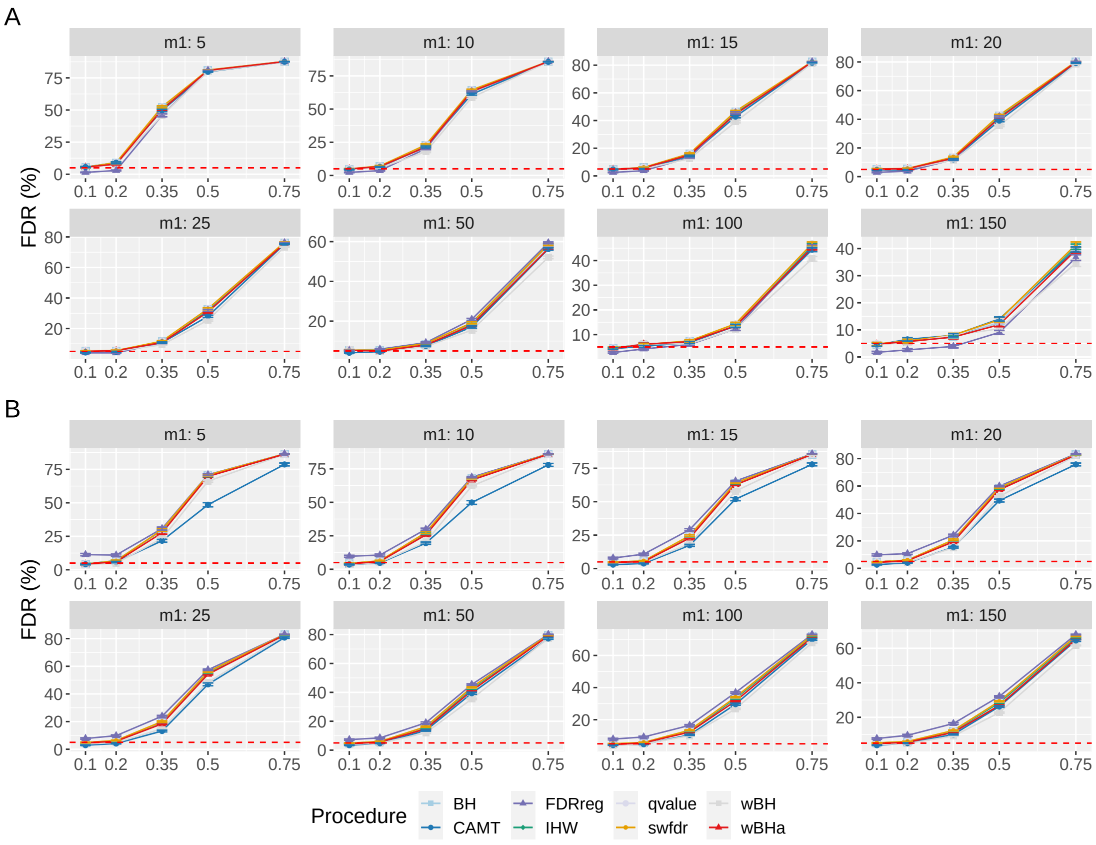

Figure S17. FDR comparison in scenario 2, with correlated markers, for different ρ and m_1 values with $m = 8000$. Panels A and B display results for quantitative and binary traits, respectively. Red dashed line corresponds to target FDR level (5%). Vertical bars illustrate standard errors.

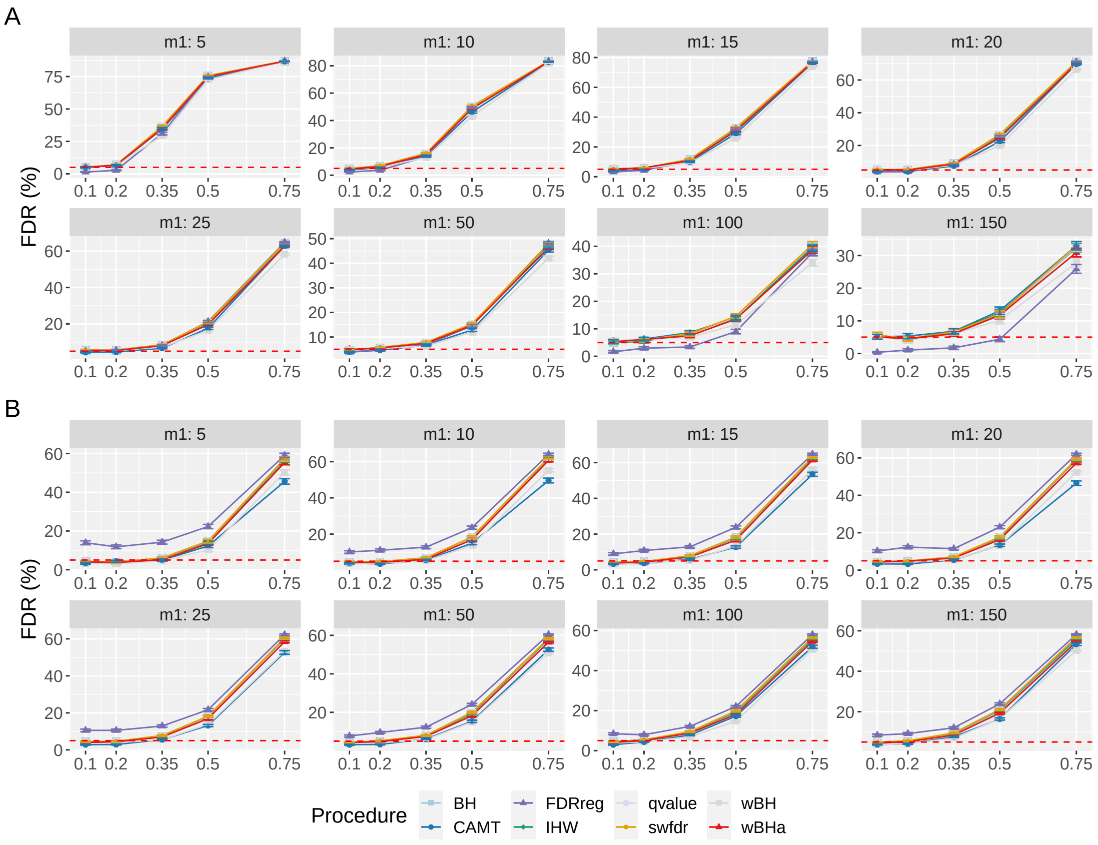

Figure S18. FDR comparison in scenario 3, with correlated markers, for different ρ and m_1 values with $m = 8000$. Panels A and B display results for quantitative and binary traits, respectively. Red dashed line corresponds to target FDR level (5%). Vertical bars illustrate standard errors.

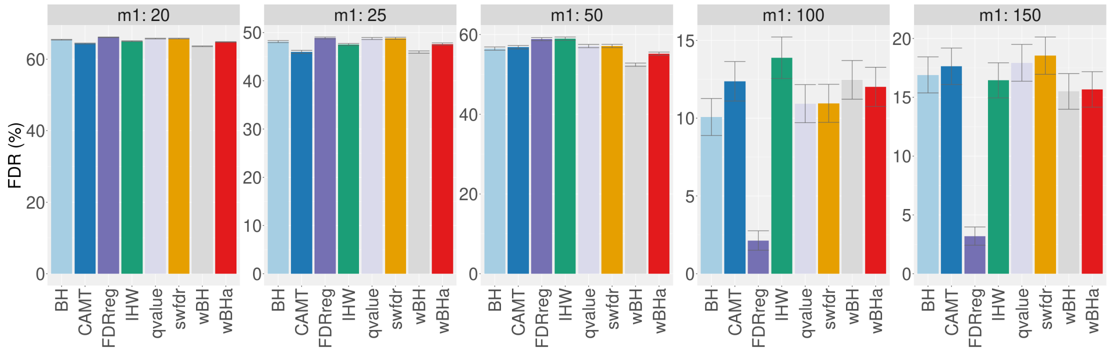

Figure S19. FDR comparison in scenario 1, with simulations based on real data, for different m_1 values. Vertical bars illustrate standard errors.

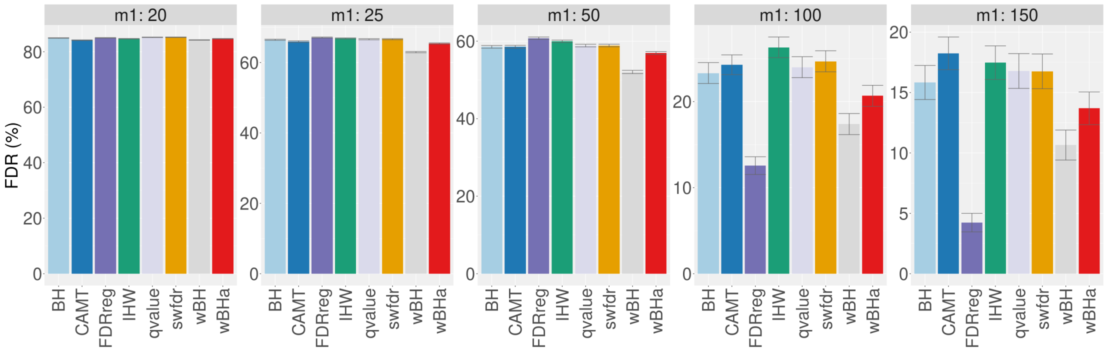

Figure S20. FDR comparison in scenario 2, with simulations based on real data, for different m_1 values. Vertical bars illustrate standard errors.

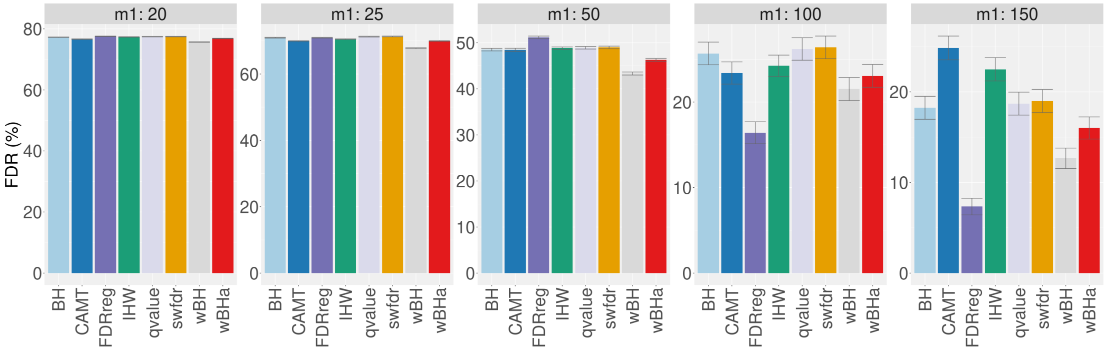

Figure S21. FDR comparison in scenario 3, with simulations based on real data, for different m_1 values. Vertical bars illustrate standard errors.

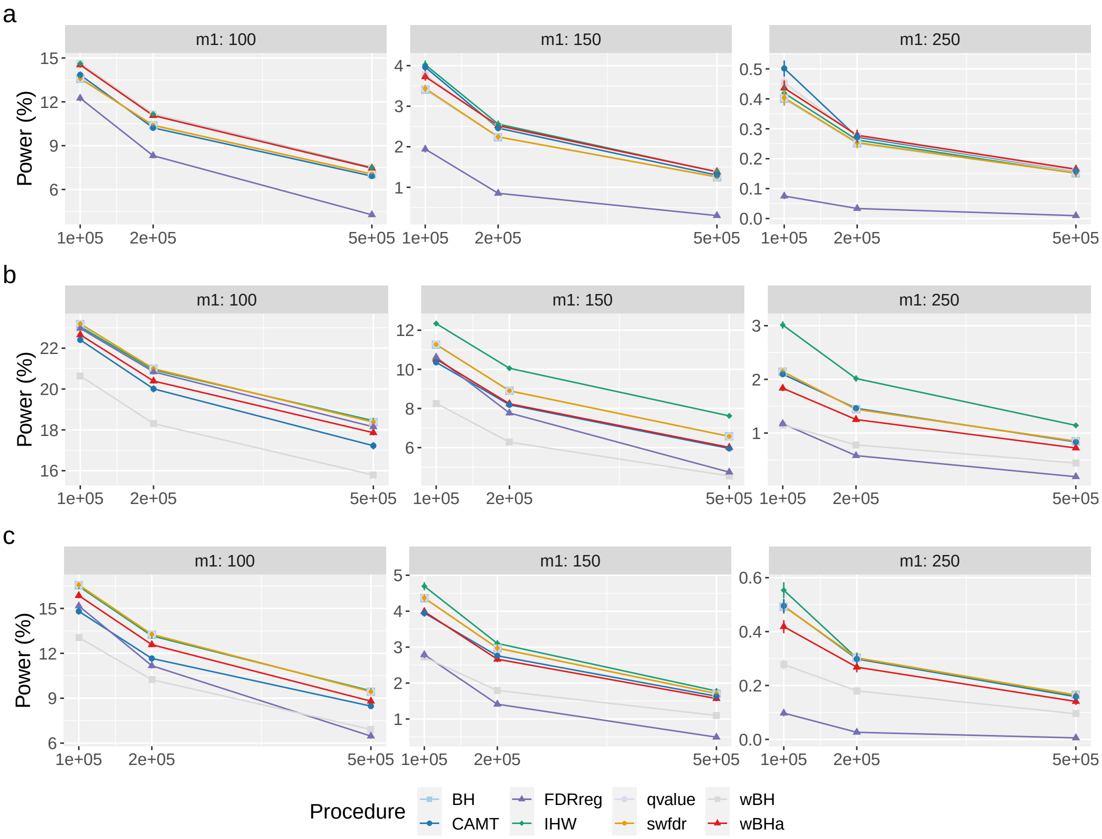

Figure S22. Overall power comparison with independent markers for **large *m*** values and m_1 values with quantitative phenotype. Panels a, b and c display results for scenario 1, scenario 2 and scenario 3, respectively. Vertical bars illustrate standard errors.

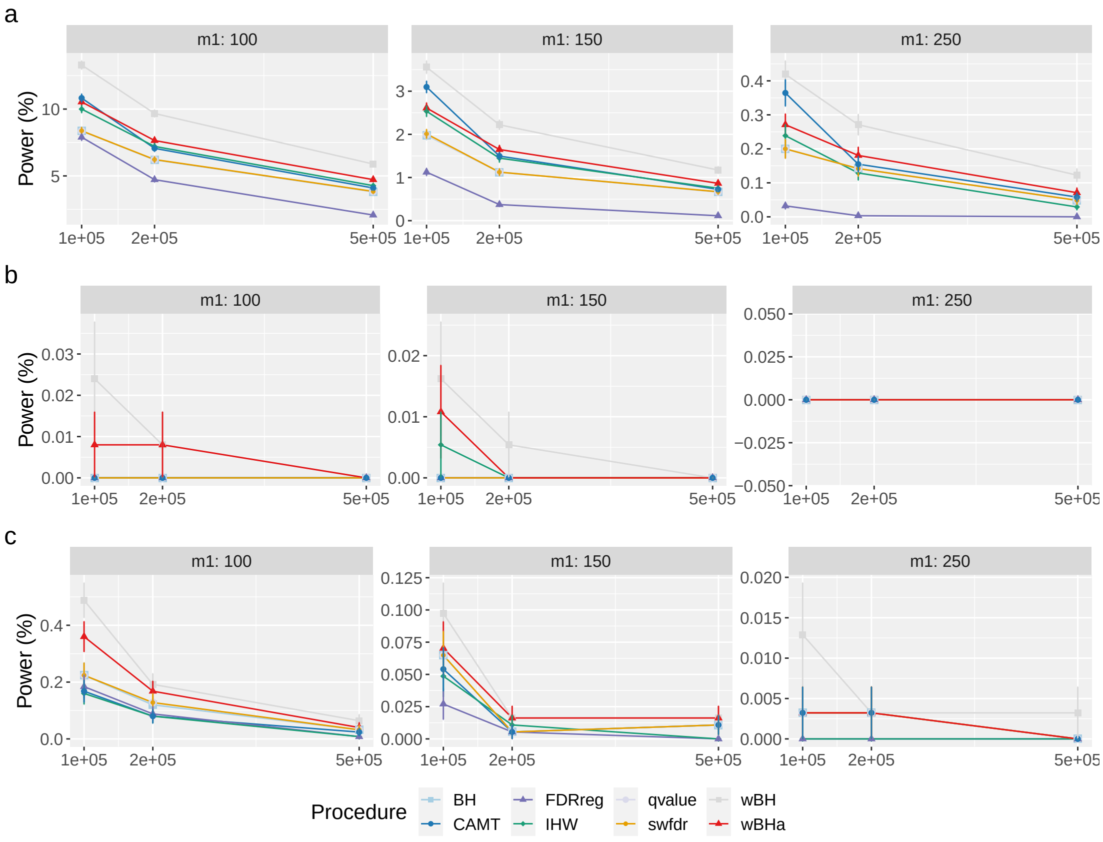

Figure S23. Power comparison in subgroup of **rare variants** with independent markers for **large *m*** values and m_1 values with quantitative phenotype. Panels a, b and c display results for scenario 1, scenario 2 and scenario 3, respectively. Vertical bars illustrate standard errors.

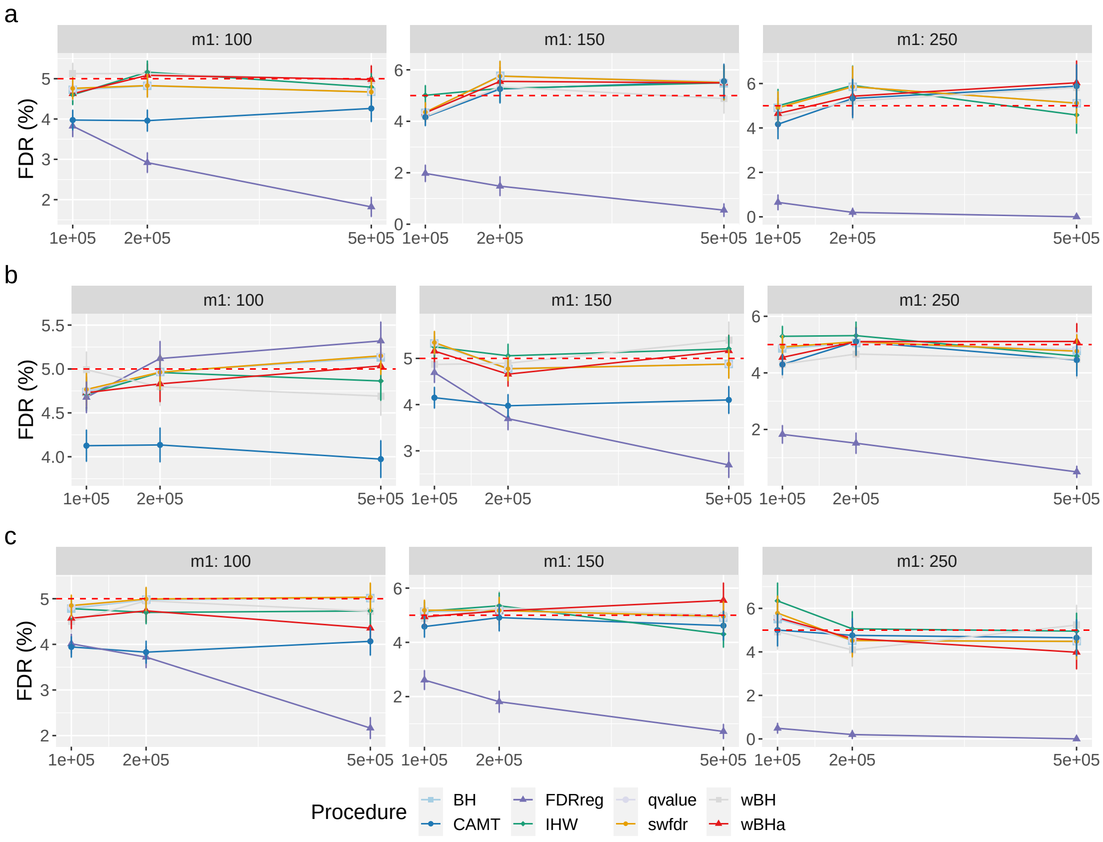

Figure S24. FDR comparison with independent markers for large m values and m_1 values with quantitative phenotype. Panels a, b and c display results for scenario 1, scenario 2 and scenario 3, respectively. Vertical bars illustrate standard errors.

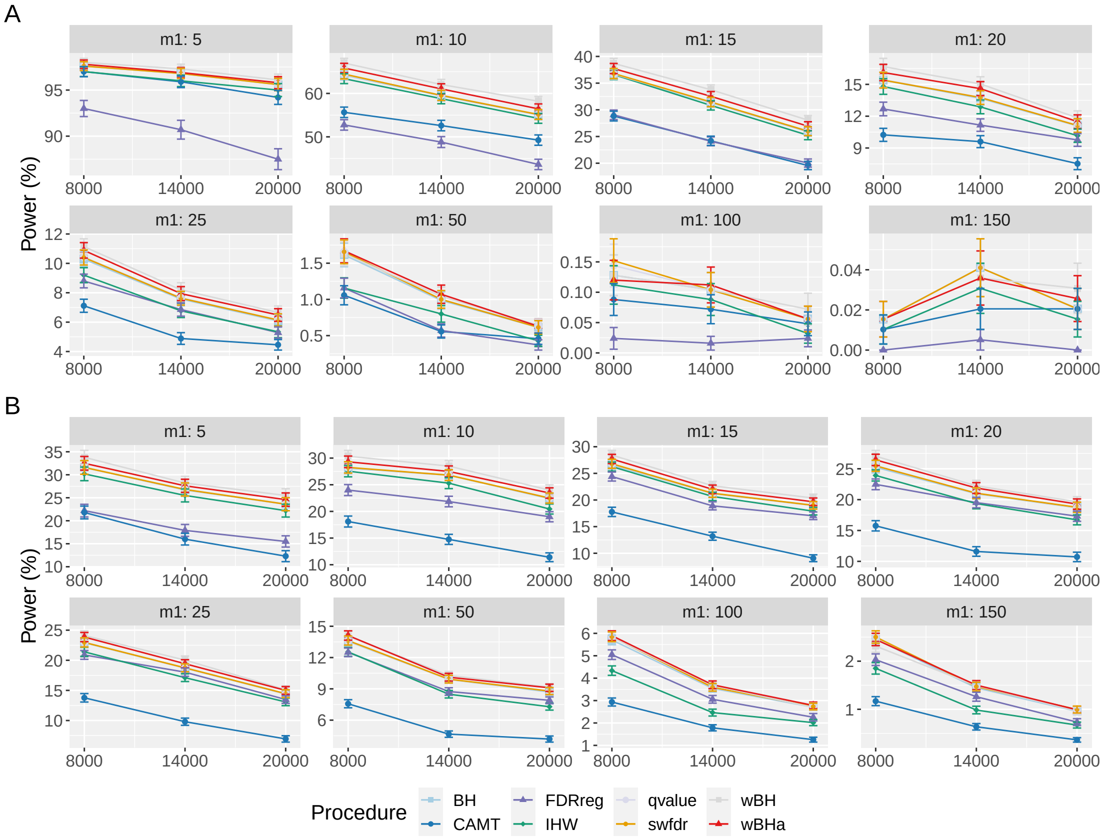

Figure S25. Power comparison in subgroup of **common variants** when using **1/MAF** as covariate in **scenario 1** with independent markers for different m and m_1 values. Panels A and B display results for quantitative and binary traits, respectively. Vertical bars illustrate standard errors.

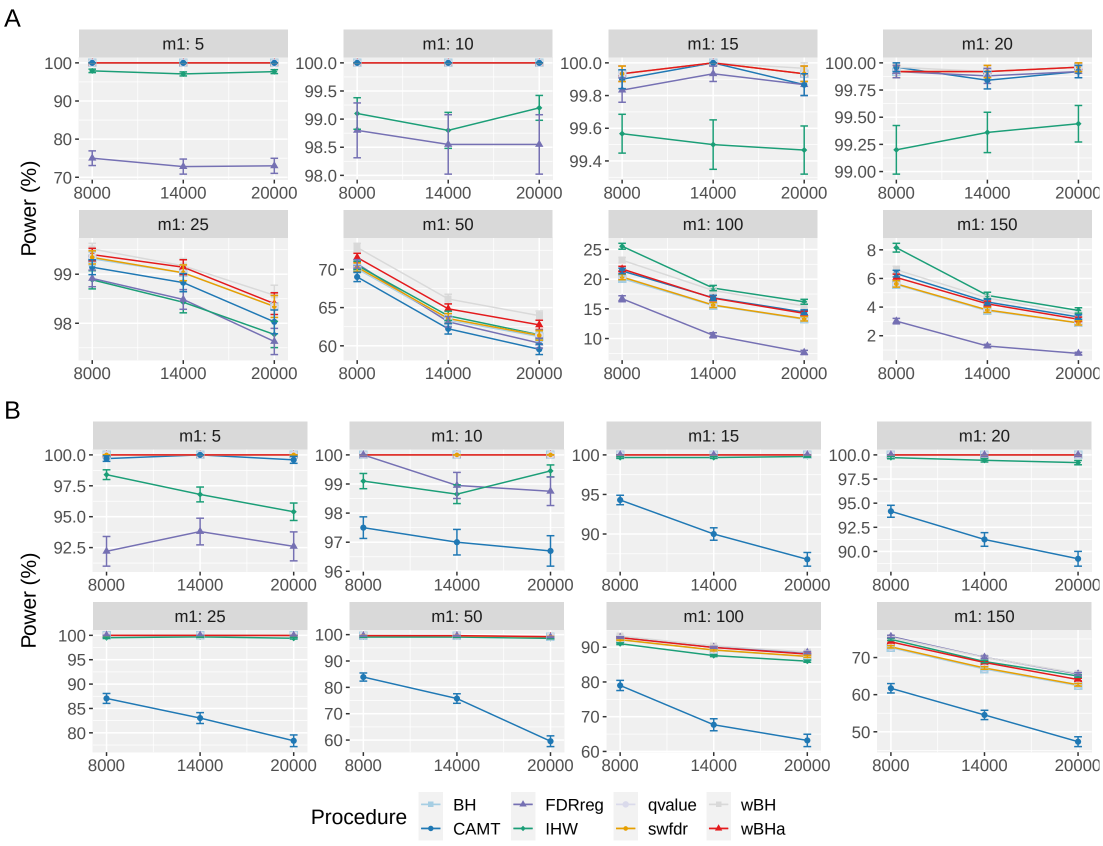

Figure S26. Power comparison in subgroup of **common variants** when using **1/MAF** as covariate in **scenario 2** with independent markers for different m and m_1 values. Panels A and B display results for quantitative and binary traits, respectively. Vertical bars illustrate standard errors.

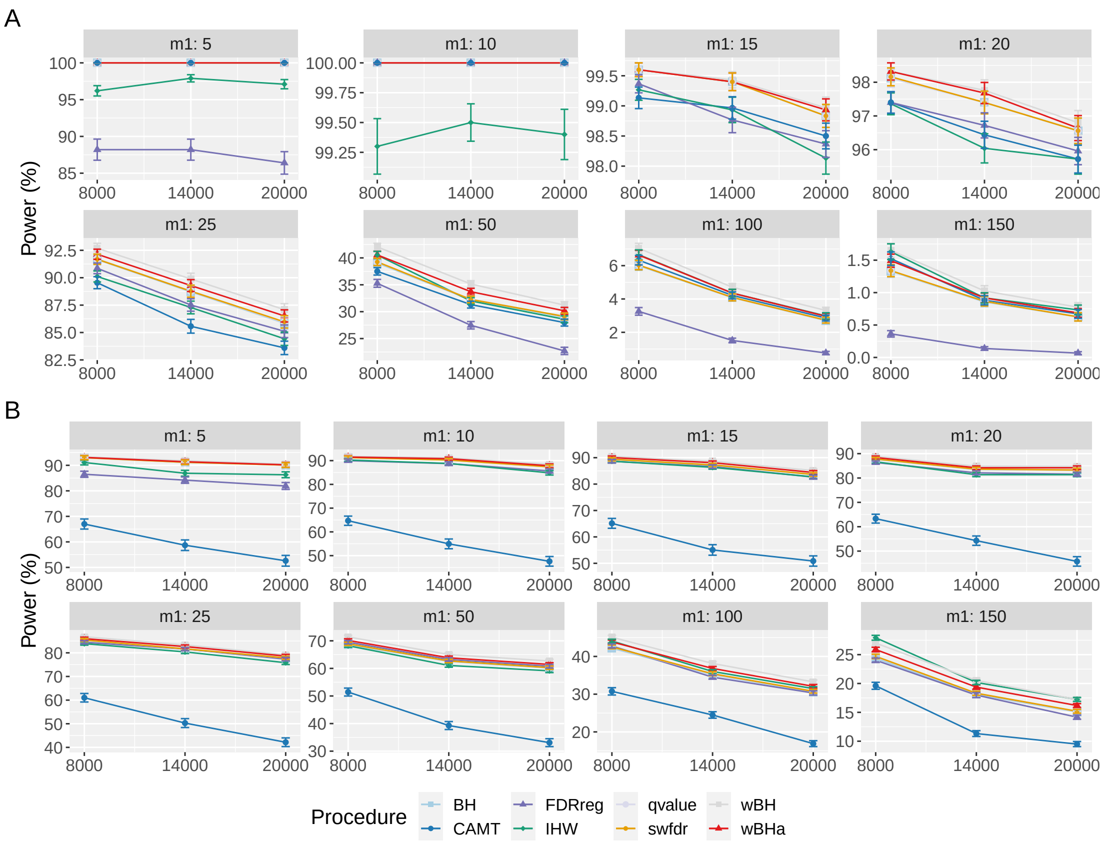

Figure S27. Power comparison in subgroup of **common variants** when using **1/MAF** as covariate in **scenario 3** with independent markers for different m and m_1 values. Panels A and B display results for quantitative and binary traits, respectively. Vertical bars illustrate standard errors.

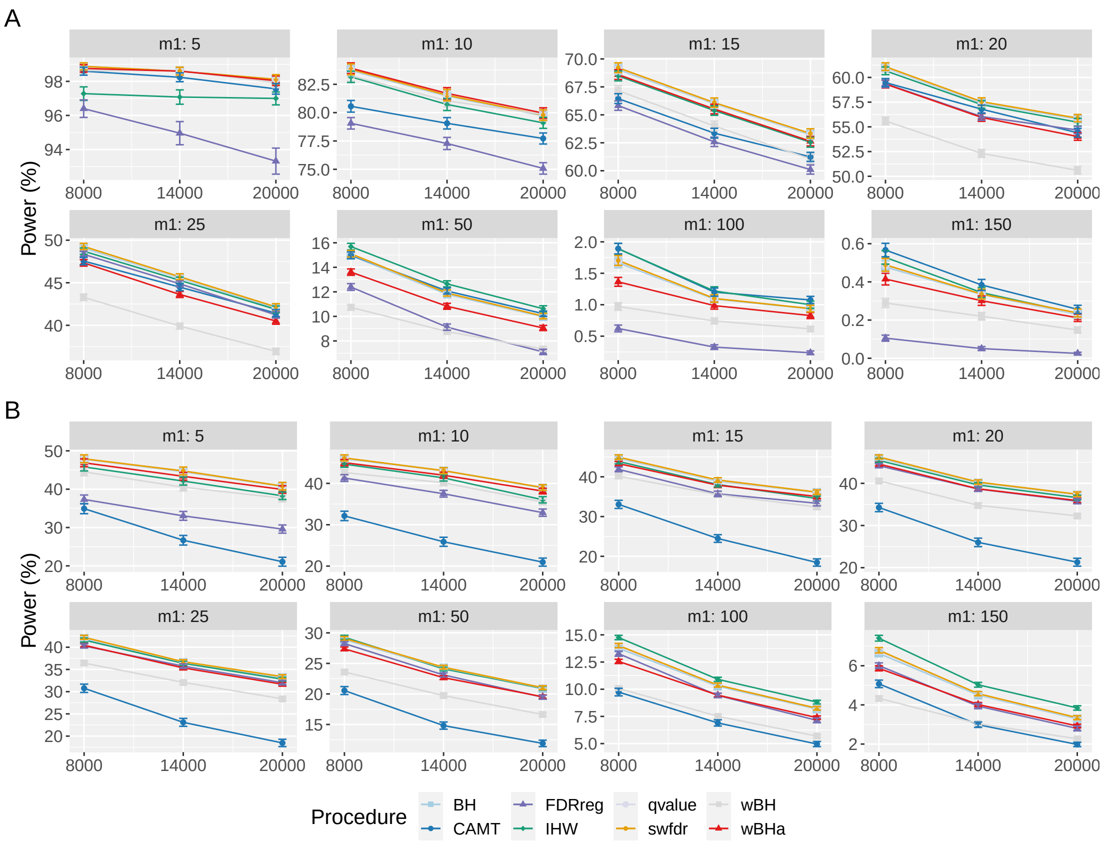

Figure S28. **Overall power** comparison when using **1/MAF** as covariate in **scenario 1** with independent markers for different m and m_1 values. Panels A and B display results for quantitative and binary traits, respectively. Vertical bars illustrate standard errors.

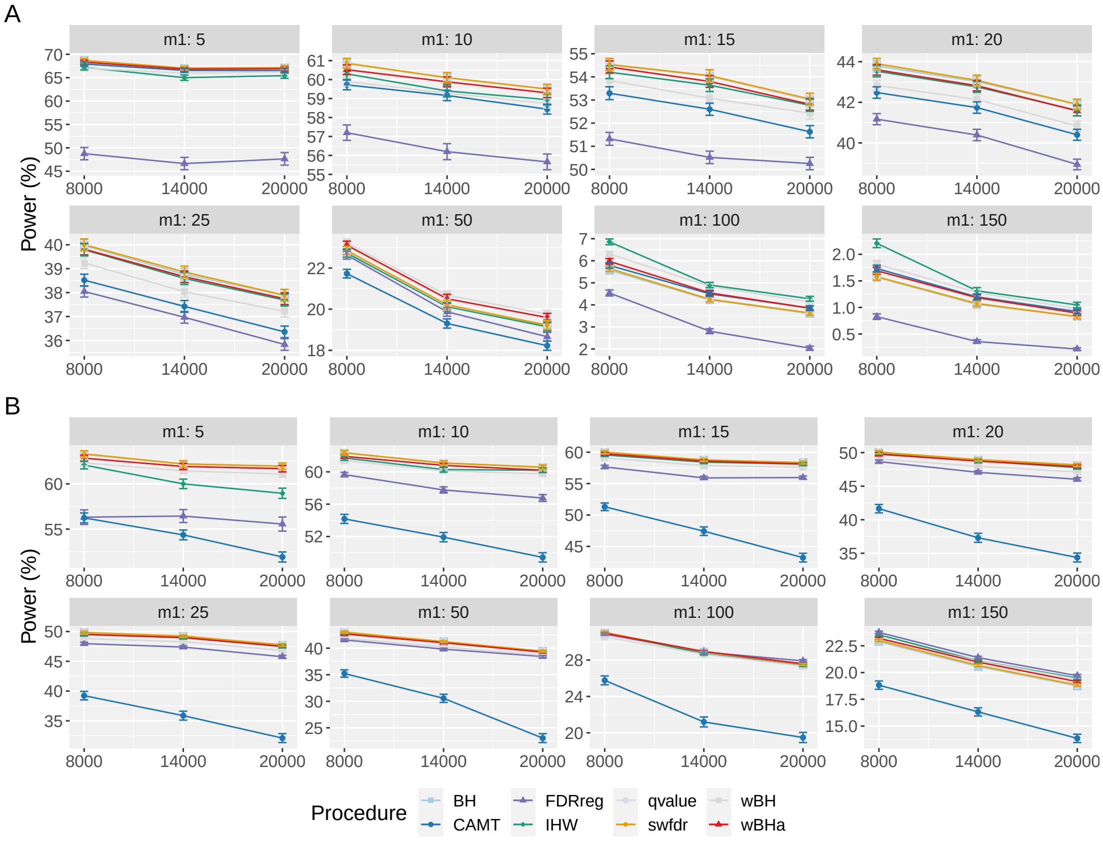

Figure S29. Overall power comparison when using 1/MAF as covariate in scenario 2 with independent markers for different m and m_1 values. Panels A and B display results for quantitative and binary traits, respectively. Vertical bars illustrate standard errors.

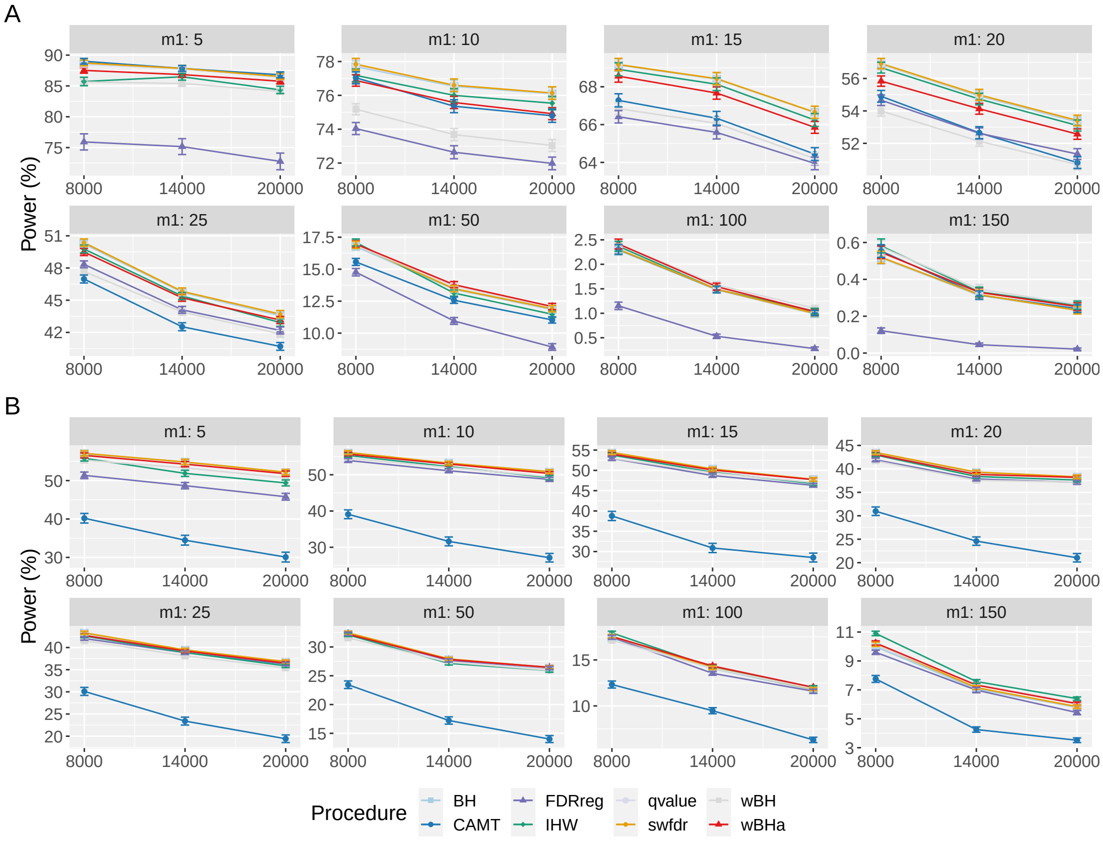

Figure S30. Overall power comparison when using 1/MAF as covariate in scenario 3 with independent markers for different m and m_1 values. Panels A and B display results for quantitative and binary traits, respectively. Vertical bars illustrate standard errors.

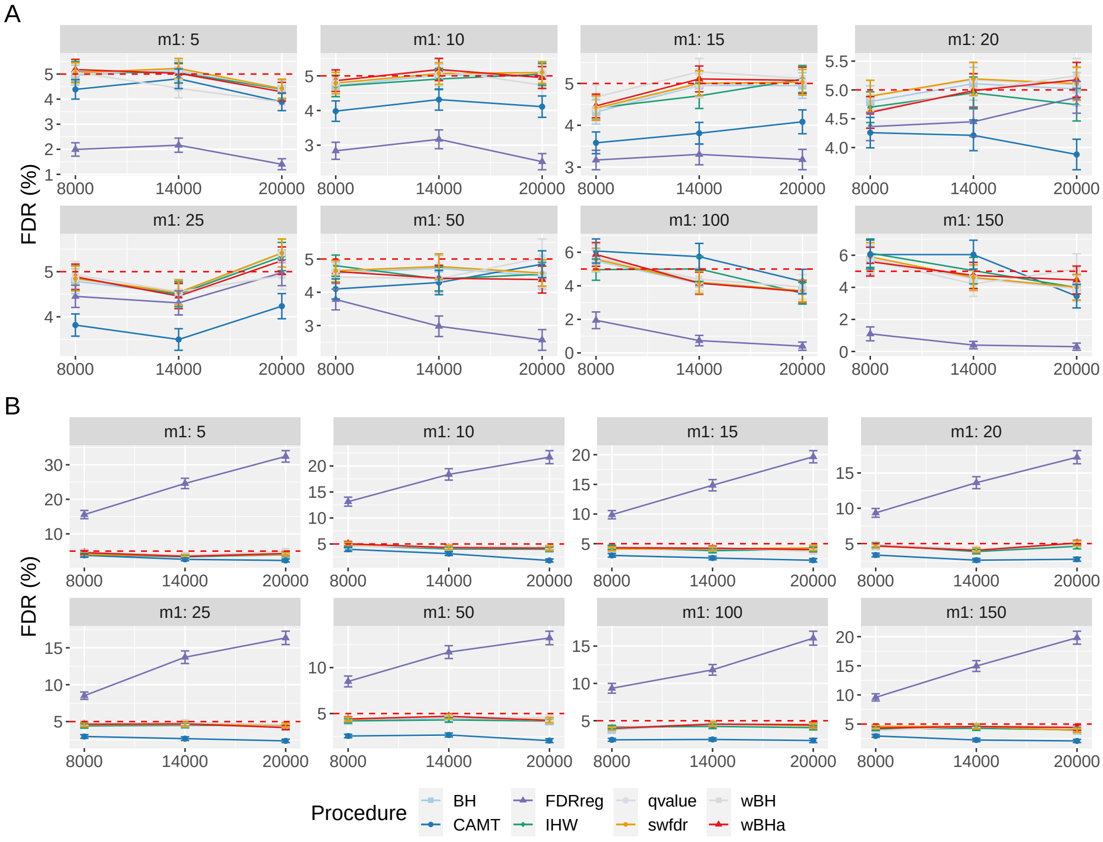

Figure S31. FDR comparison when using 1/MAF as covariate in scenario 1 with independent markers for different m and m_1 values. Panels A and B display results for quantitative and binary traits, respectively. Vertical bars illustrate standard errors.

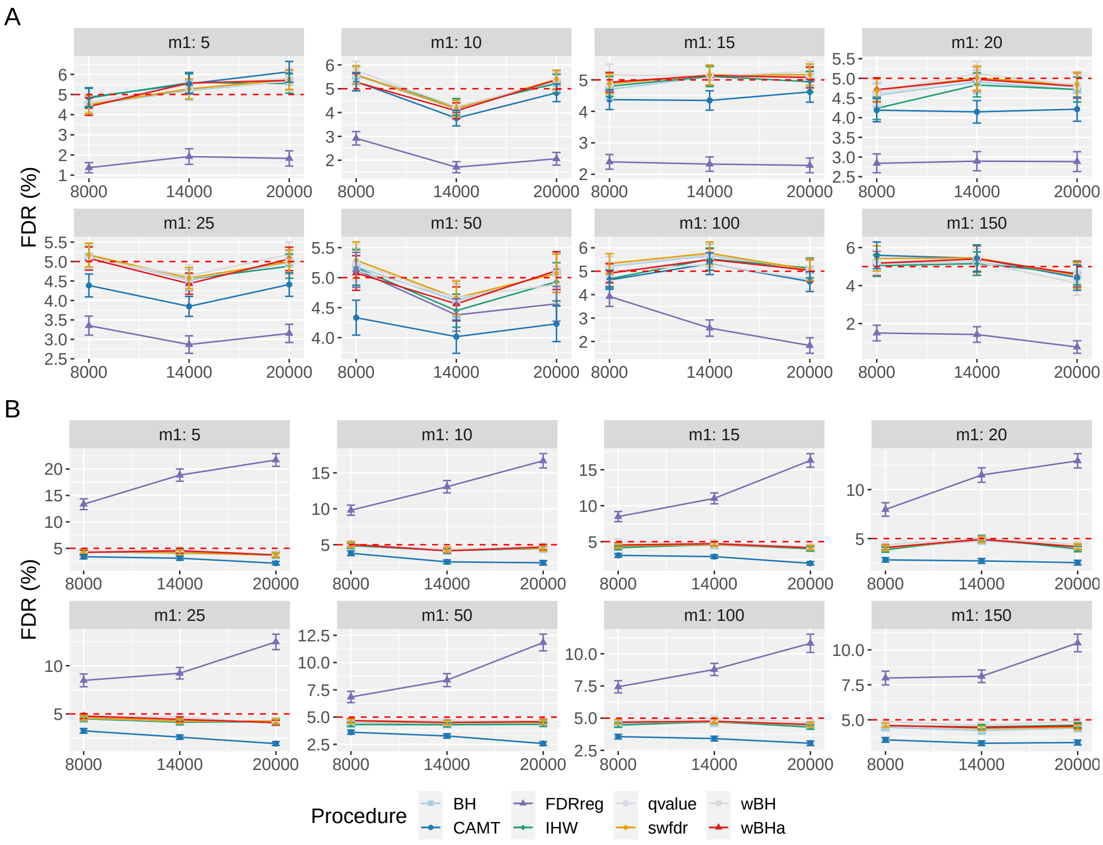

Figure S32. **FDR** comparison when using **1/MAF** as covariate in **scenario 2** with independent markers for different m and m_1 values. Panels A and B display results for quantitative and binary traits, respectively. Vertical bars illustrate standard errors.

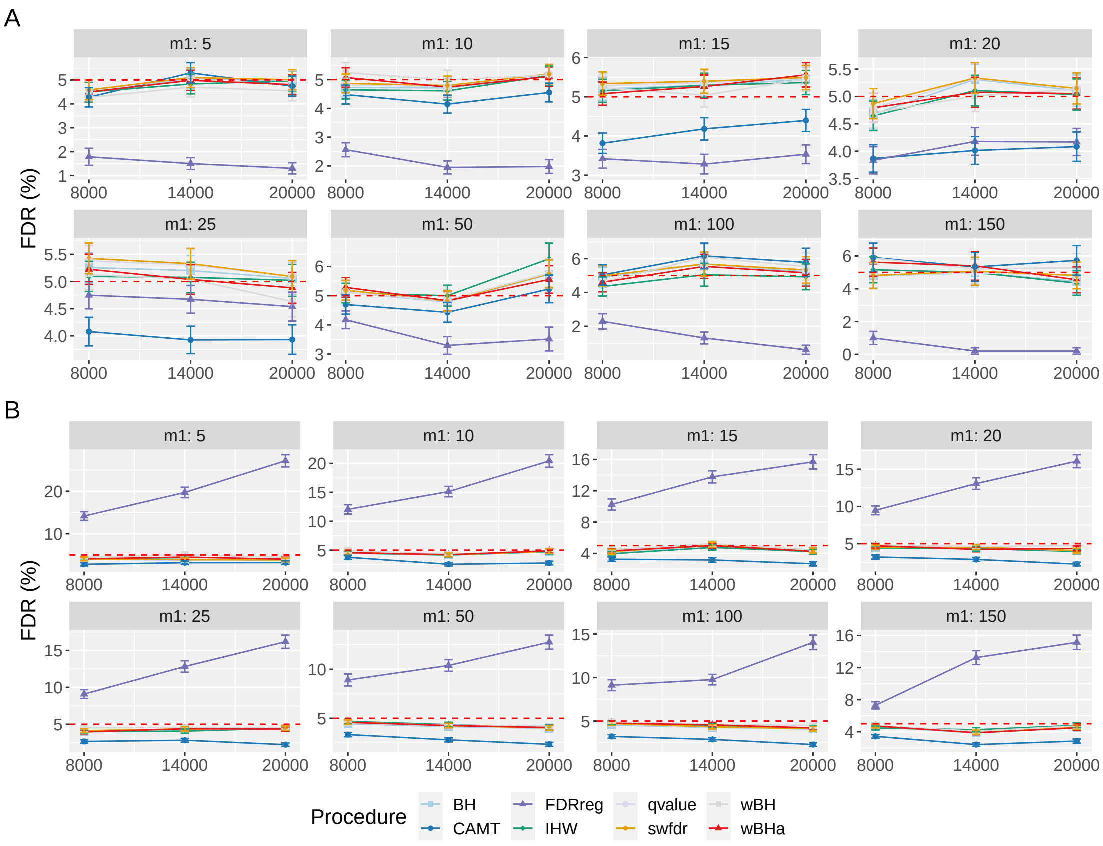

Figure S33. FDR comparison when using 1/MAF as covariate in scenario 3 with independent markers for different m and m_1 values. Panels A and B display results for quantitative and binary traits, respectively. Vertical bars illustrate standard errors.

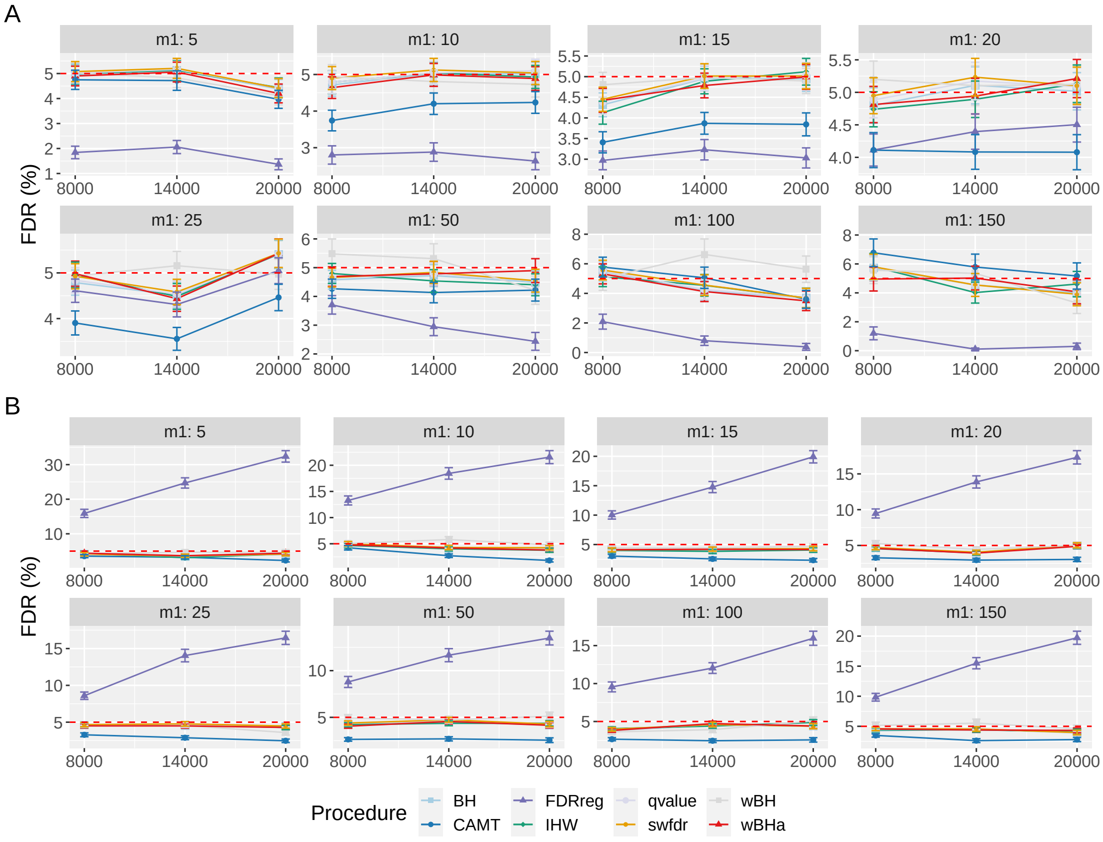

Figure S34. FDR comparison when using uninformative covariates in scenario 1 with independent markers for different m and m_1 values. Panels A and B display results for quantitative and binary traits, respectively. Vertical bars illustrate standard errors.

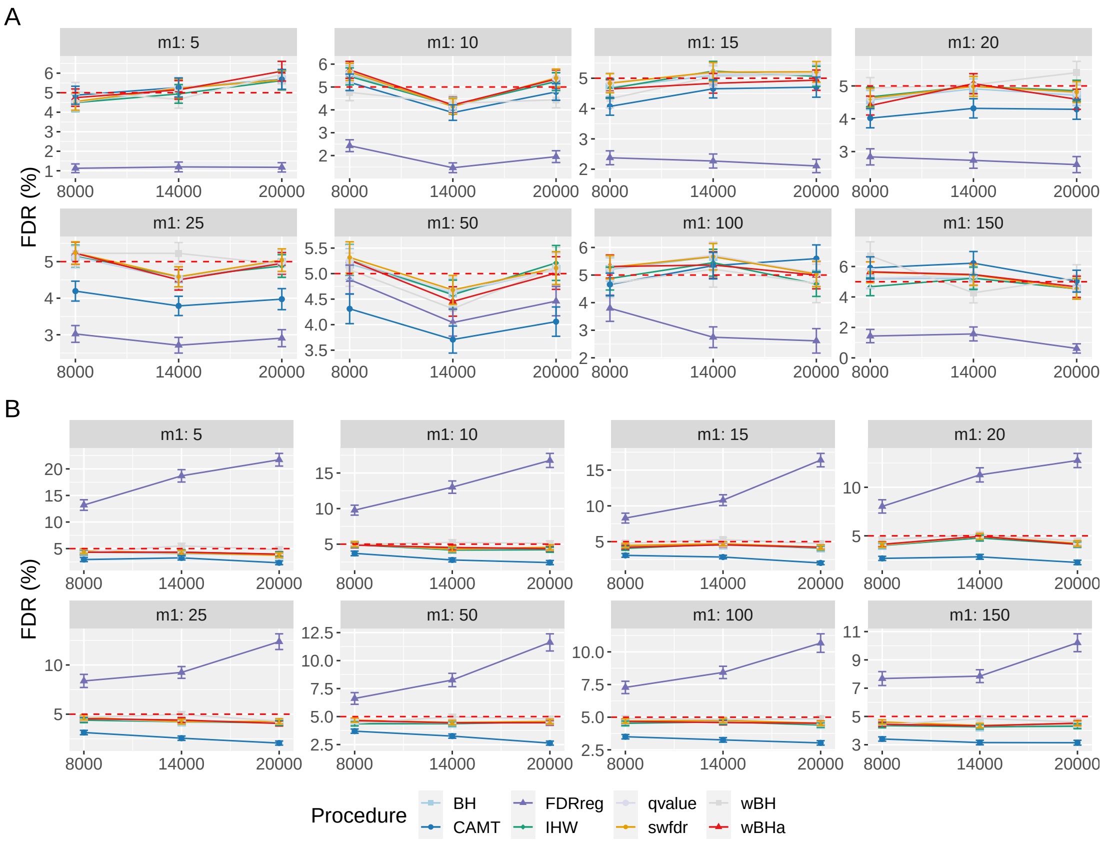

Figure S35. **FDR** comparison when using **uninformative** covariates in **scenario 2** with independent markers for different m and m_1 values. Panels A and B display results for quantitative and binary traits, respectively. Vertical bars illustrate standard errors.

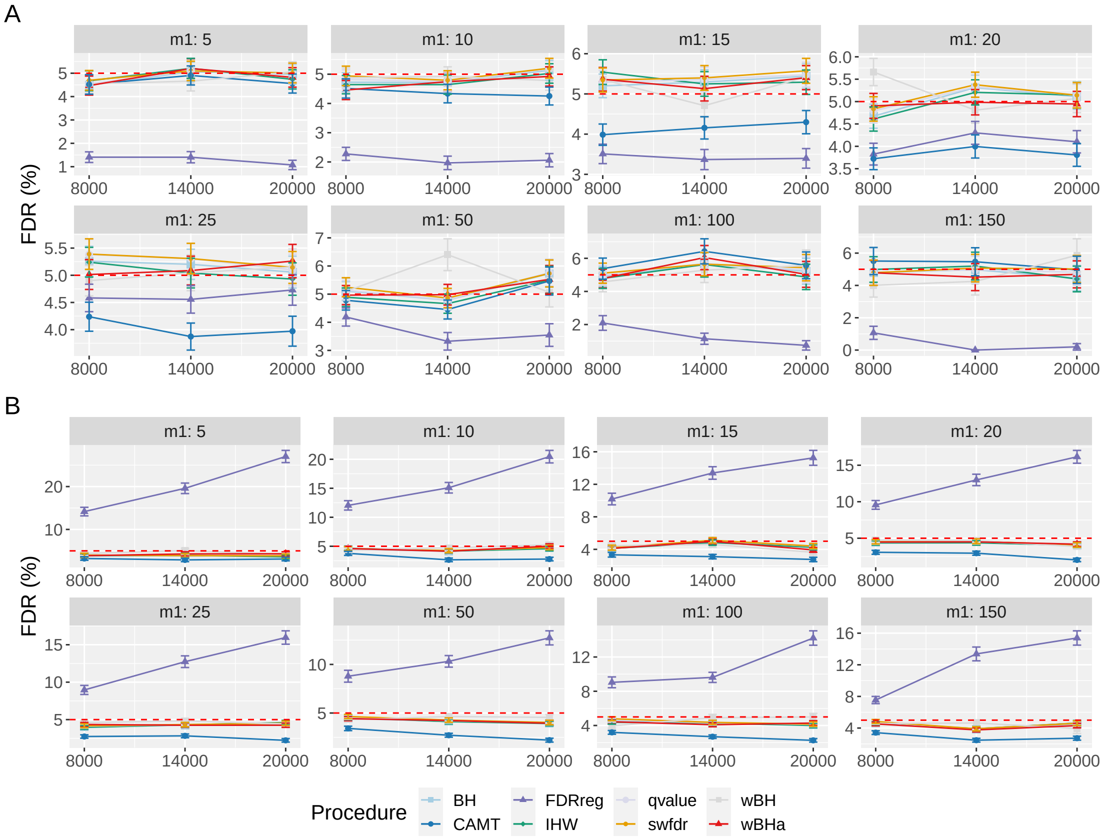

Figure S36. **FDR** comparison when using **uninformative** covariates in **scenario 3** with independent markers for different m and m_1 values. Panels A and B display results for quantitative and binary traits, respectively. Vertical bars illustrate standard errors.

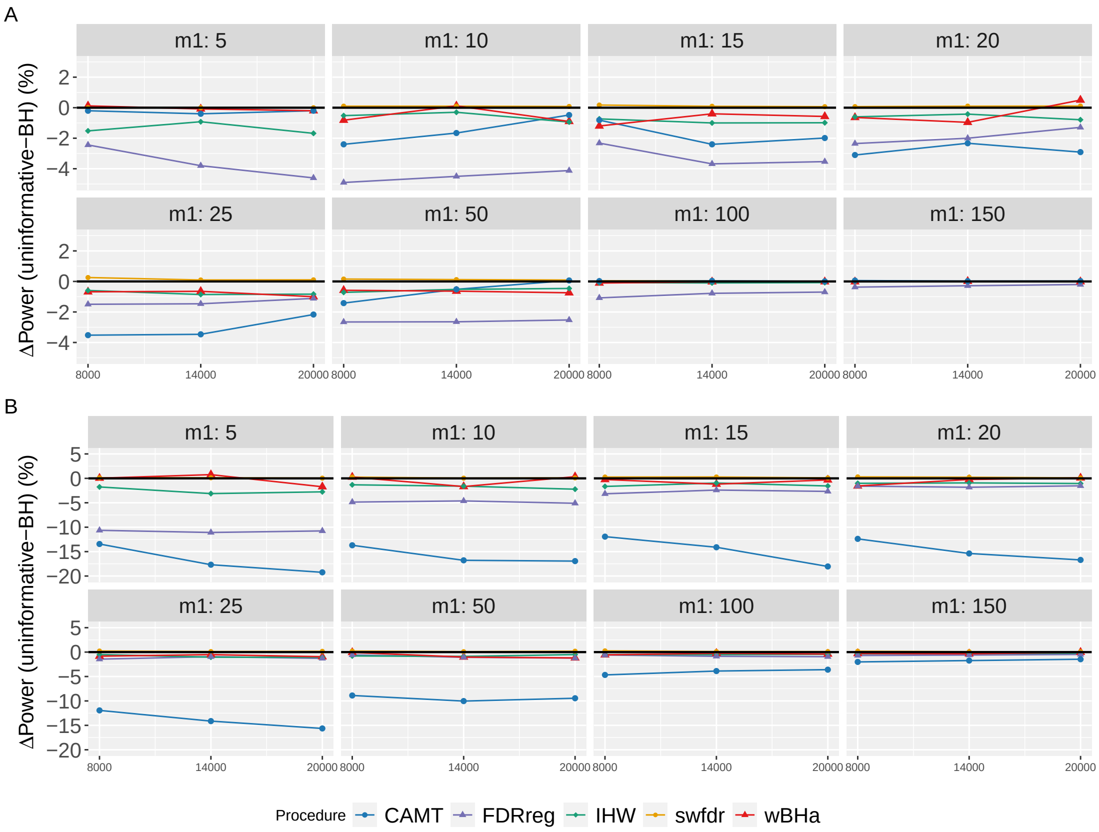

Figure S37. **Overall power difference** between uninformative covariates and BH procedure in **scenario 1** with independent markers for different m and m_1 values. Panels A and B display results for quantitative and binary traits, respectively.

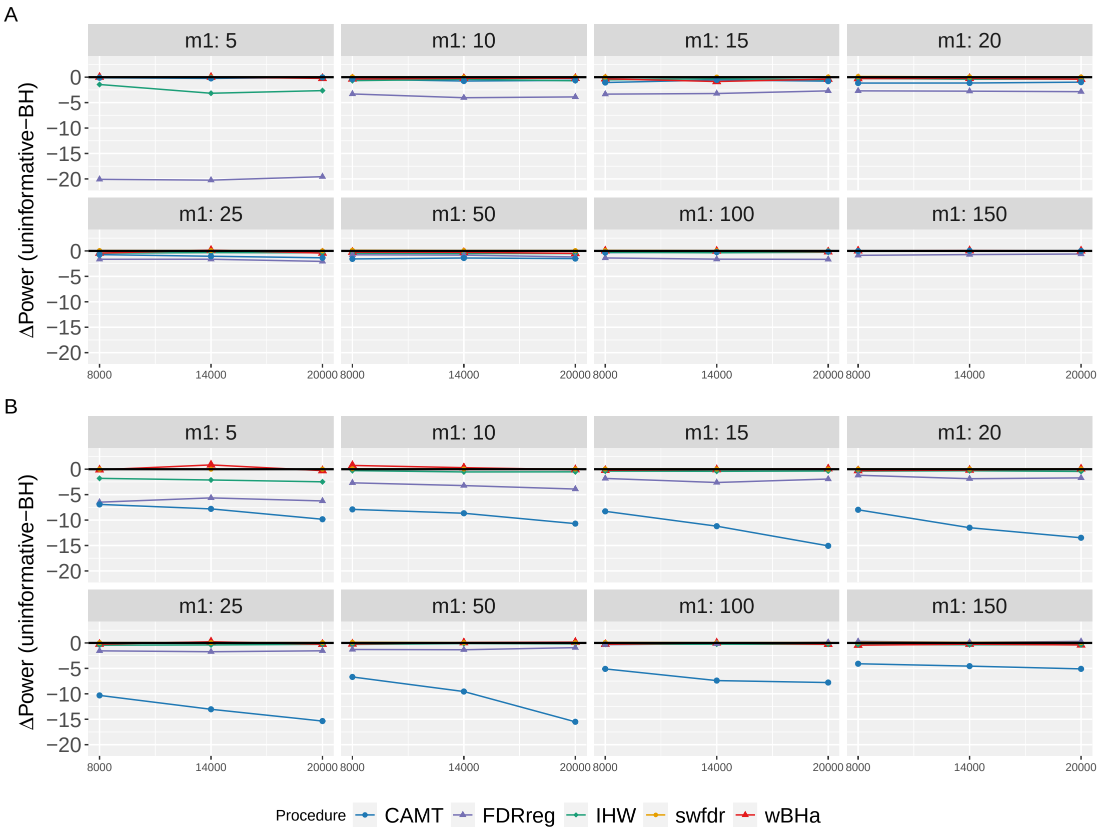

Figure S38. **Overall power difference** between uninformative covariates and BH procedure in **scenario 2** with independent markers for different m and m_1 values. Panels A and B display results for quantitative and binary traits, respectively.

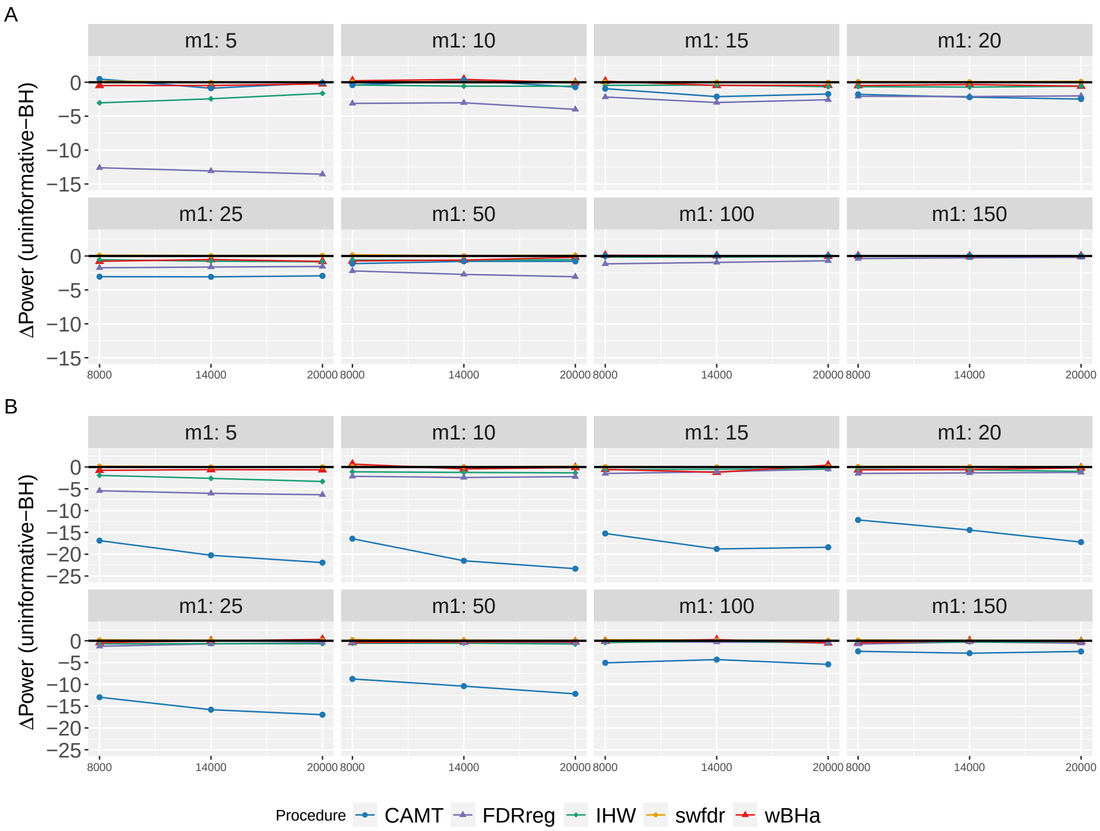

Figure S39. **Overall power difference** between uninformative covariates and BH procedure in **scenario 3** with independent markers for different m and m_1 values. Panels A and B display results for quantitative and binary traits, respectively.

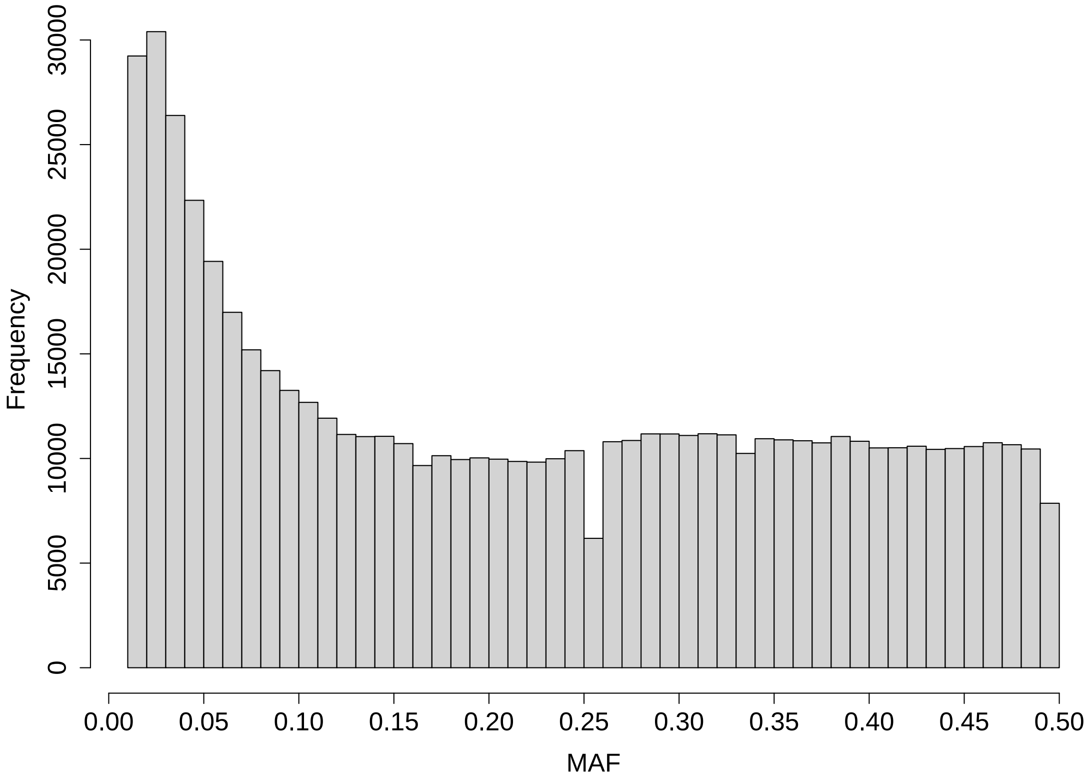

Figure S40. MAF distribution of all SNPs for the Crohn's disease dataset.
